# Synopsis of *Cis* Latreille (Coleoptera: Ciidae) from southern Africa

**DOI:** 10.3390/insects9040184

**Published:** 2018-12-05

**Authors:** Igor Souza-Gonçalves, Artur Orsetti, Cristiano Lopes-Andrade

**Affiliations:** 1 Programa de Pós-Graduação em Ecologia Departamento de Biologia Geral, Universidade Federal de Viçosa, Viçosa 36570-900, Brazil; 2 Laboratório de Sistemática e Biologia de Coleoptera, Departamento de Biologia Animal, Universidade Federal de Viçosa, Viçosa 36570-900, Brazil; arturorsetti@gmail.com (A.O.); ciidae@gmail.com (C.L.-A.); 3 Programa de Pós-Graduação em Biologia Animal, Departamento de Biologia Animal, Universidade Federal de Viçosa, Viçosa 36570-900, Brazil

**Keywords:** minute tree-fungus beetles, Ciinae, Ciini, sub-Saharan, Ethiopian, identification key

## Abstract

A synopsis of the *Cis* Latreille, 1796 from southern Africa is provided, with the description of 10 new species: *Cis bicaesariatus* **sp. n.**, *Cis foveocephalus* **sp. n.**, *Cis grobbelaarae* **sp. n.**, *Cis lacinipennis* **sp. n.**, *Cis makrosoma* **sp. n.**, *Cis mpumalangaensis* **sp. n.**, *Cis parvisetosus* **sp. n.**, *Cis tessariplacus* **sp. n.**, *Cis umlalaziensis* **sp. n.** and *Cis westerncapensis* **sp. n.** The introduced species *Cis fuscipes* Mellié, 1849 is recorded for the first time from the Republic of South Africa. New geographic records are provided for the following species: *Cis neserorum* Souza-Gonçalves & Lopes-Andrade, 2017; *Cis regius* Orsetti & Lopes-Andrade, 2016 and *Cis stalsi* Souza-Gonçalves & Lopes-Andrade, 2017. Most southern African *Cis* are placed in available or newly proposed species-groups and a provisional identification key is provided.

## 1. Introduction

Ciidae is a cosmopolitan family and comprises of more than 700 described species in 51 genera. The genus *Cis* Latreille, 1796 (Ciinae: Ciini) has about 400 described species occurring in all biogeographic regions, except for the Antarctic [1,2,3]. It is the most diverse genus in the family, including more than half of all described Ciidae, but it is possibly polyphyletic [4,5]. The previously proposed division into subgenera is not in use and part of the *Cis* species is organized in artificial species-group [2,3,6,7].

The sub-Saharan Ciidae, most belonging to the Ethiopian region (biogeographic regions *sensu* Morrone [8]), are represented by 75 described species [1,9,10,11,12], of which 53 belong to *Cis*. In southern Africa, comprising Botswana, Lesotho, Namibia, Republic of South Africa, Swaziland and the southern tip of Mozambique, there are 19 described species of *Cis*: *C. afer* Fåhraeus, 1871, *C. aster* Souza-Gonçalves & Lopes-Andrade, 2017, *C. bimucronatus* Motschoulsky, 1851, *C. caffer* Fåhraeus, 1871, *C. capensis* Mellié, 1849, *C. chinensis* Lawrence, 1991, *C. delagoensis* Pic, 1916, *C. makebae* Souza-Gonçalves & Lopes-Andrade, 2017, *C. mandelai* Souza-Gonçalves & Lopes-Andrade, 2017, *C. masekelai* Souza-Gonçalves-& Lopes-Andrade, 2017, *C. mooihoekite* Souza-Gonçalves & Lopes-Andrade, 2018, *C. muriceus* Mellié, 1849, *C. neserorum* Souza-Gonçalves & Lopes-Andrade, 2017, *C. paraliacus* Souza-Gonçalves & Lopes-Andrade, 2018, *C. pickeri* Lopes-Andrade et al., 2009, *C. regius* Orsetti & Lopes-Andrade, 2016, *C. stalsi* Souza-Gonçalves & Lopes-Andrade, 2017, *C. testaceus* Fåhraeus, 1871 and *C. urbanae* Souza-Gonçalves & Lopes-Andrade, 2017.

The aim of this paper is to provide a synopsis of southern African *Cis*, with description of 10 new species. Most of them are placed in available or newly proposed species-groups. New geographic records for previously described species are provided, together with a provisional identification key to species occurring in southern Africa. The invasive *Cis fuscipes* Mellié, 1849 is recorded for the first time from the Republic of South Africa.

## 2. Materials and Methods

Museum abbreviations are as follows:

**ANIC**  Australian National Insect Collection, CSIRO Entomology (Canberra, Australian Capital Territory, Australia)
**CELC**  Coleção Entomológica do Laboratório de Sistemática e Biologia de Coleoptera da Universidade Federal de Viçosa (Viçosa, Minas Gerais, Brazil)
**CUIC**  Cornell University Insect Collection (Ithaca, NY, USA)
**SANC**  South African National Collection of Insects (Pretoria, Gauteng, Republic of South Africa)

**Figure 1:**
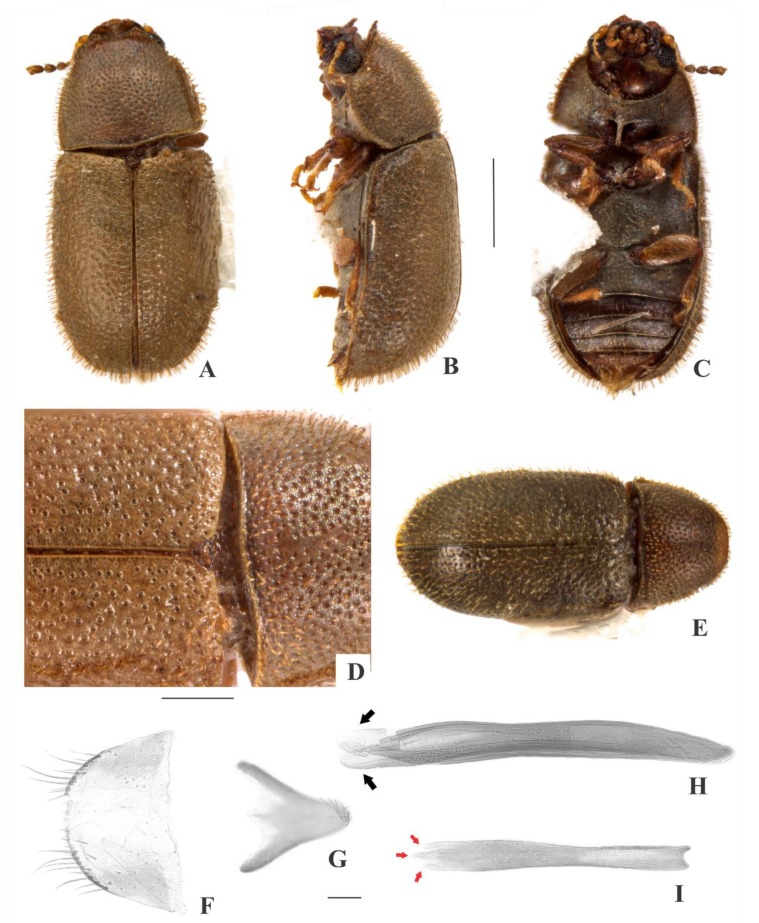
*Cis bicaesariatus***sp. n.**, male holotype (**A**–**D**), female paratype (**E**), aedeagus of holotype (**F**–**I**): **A.** Dorsal view. **B.** Lateral view. **C.** Ventral view. **D.** Scutellar shield and part of the pronotum and elytra. **F.** Sternite VIII. **G.** Basal piece. **H.** Tegmen, note bilobed apex (black arrows). **I.** Penis, note acute angulatios at apex (red arrows). Scale bars: 0.5 mm (**A**–**C**,**E**), 0.2 mm (**D**), 0.05 mm (**F–I**).

**Figure 2:**
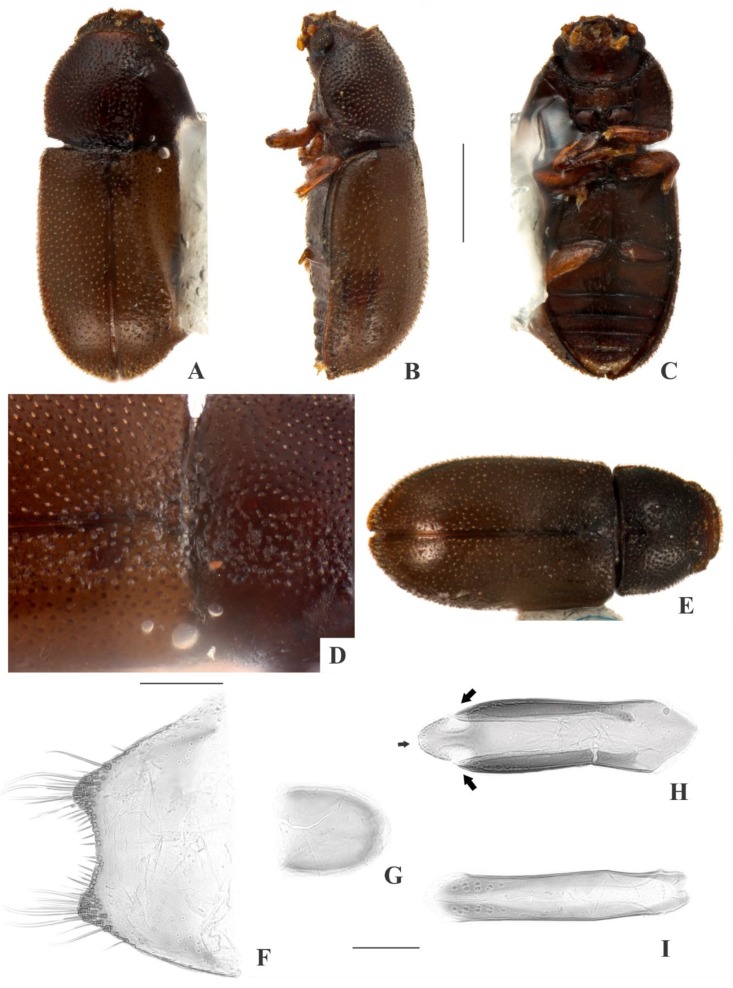
*Cis foveocephalus***sp. n.**, male holotype (**A**–**D**), female paratype (**E**), aedeagus of paratype from type locality (**F**–**I**): (**A**) Dorsal view. (**B**) Lateral view. (**C**) Ventral view. (**D**) Scutellar shield and part of the pronotum and elytra. (**F**) Sternite VIII. (**G**) Basal piece. (**H**) Tegmen, note acute lobes (big black arrows) and arrow-shaped lobe (small black arrow) at apex. (**I**) Penis. Scale bars: 0.5 mm (**A**–**C**,**E**), 0.2 mm (**D**), 0.05 mm (**F**–**I**).

**Figure 3:**
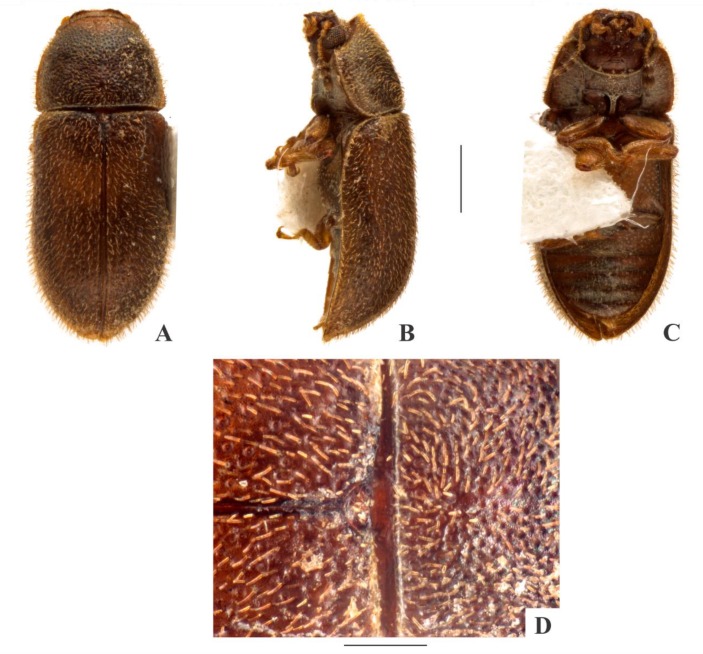
*Cis fuscipes* Mellié, 1849, female (**A**–**D**): (**A**) Dorsal view. (**B**) Lateral view. (**C**) Ventral view. (**D**) Scutellar shield and part of the pronotum and elytra. Scale bars: 0.5 mm (**A**–**C**), 0.2 mm (**D**).

**Figure 4:**
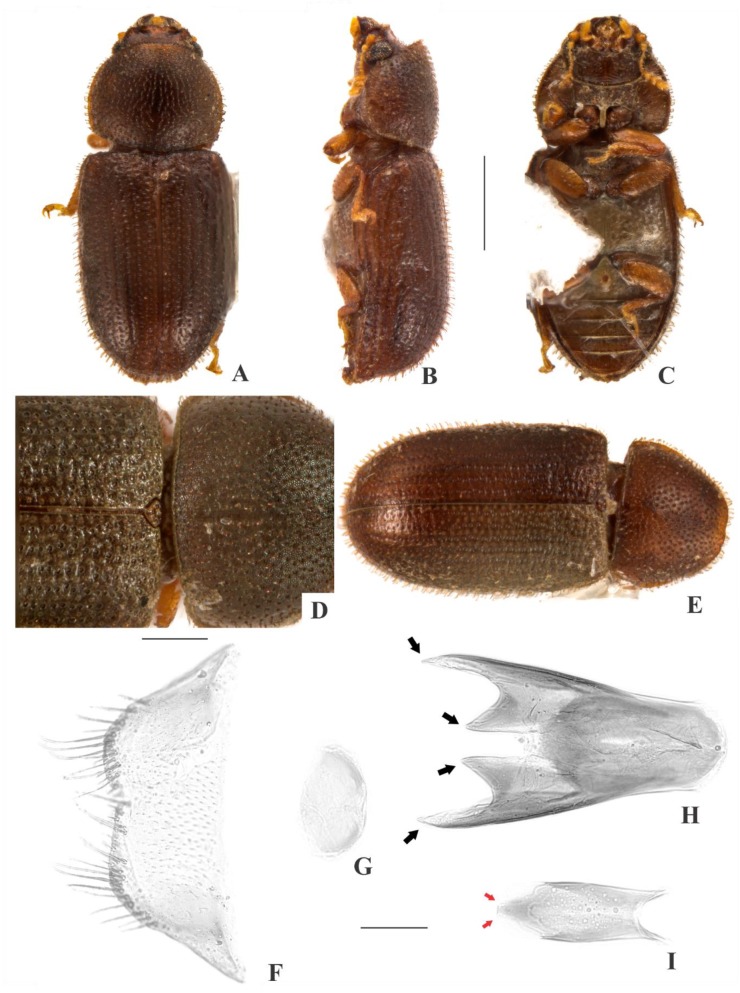
*Cis grobbelaarae***sp. n.**, male holotype (**A**–**D**), female paratype (**E**), aedeagus of paratype from type locality (**F**–**I**): (**A**) Dorsal view. (**B**) Lateral view. (**C**) Ventral view. (**D**) Scutellar shield and part of the pronotum and elytra. (**F**) Sternite VIII. (**G**) Basal piece. (**H**) Tegmen, note angulations at apical corners (black arrows). (**I**) Penis, note truncate apex (red arrows). Scale bars: 0.5 mm (**A**–**C**,**E**), 0.2 mm (**D**), 0.05 mm (**F**–**I**).

**Figure 5:**
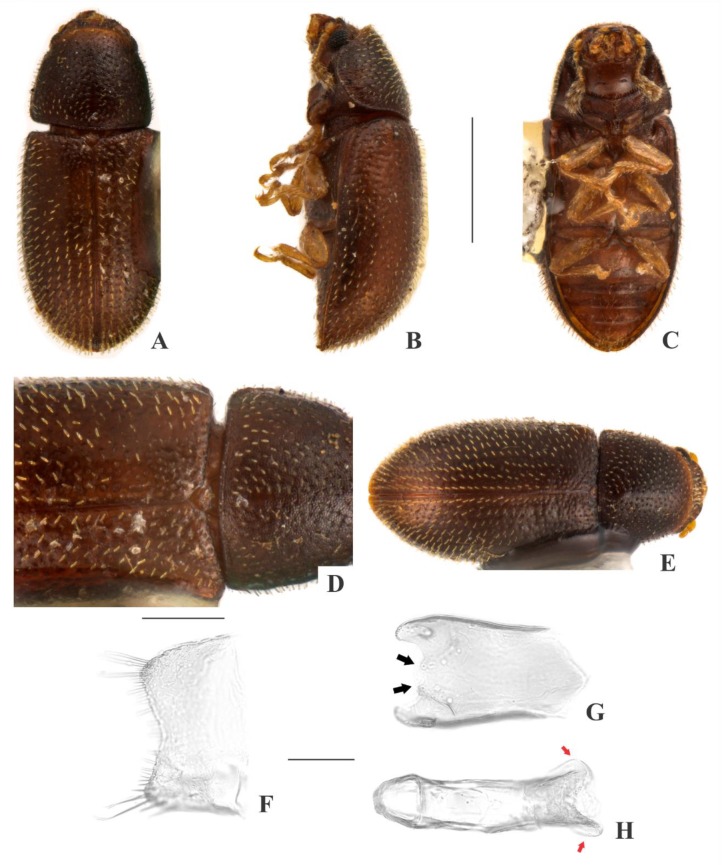
*Cis lacinipennis***sp. n.**, male holotype (**A**–**D**), female paratype (**E**), aedeagus of paratype from type locality (**F**–**H**): (**A**) Dorsal view. (**B**) Lateral view. (**C**) Ventral view. (**D**) Scutellar shield and part of the pronotum and elytra. (**F**) Sternite VIII. (**G**) Tegmen, note small tubercles at apex (black arrows). (**H**) Penis, note flaps at anterior portion (red arrows). Scale bars: 0.5 mm (**A**–**C**, **E**), 0.2 mm (**D**), 0.05 mm (**F**–**H**).

**Figure 6:**
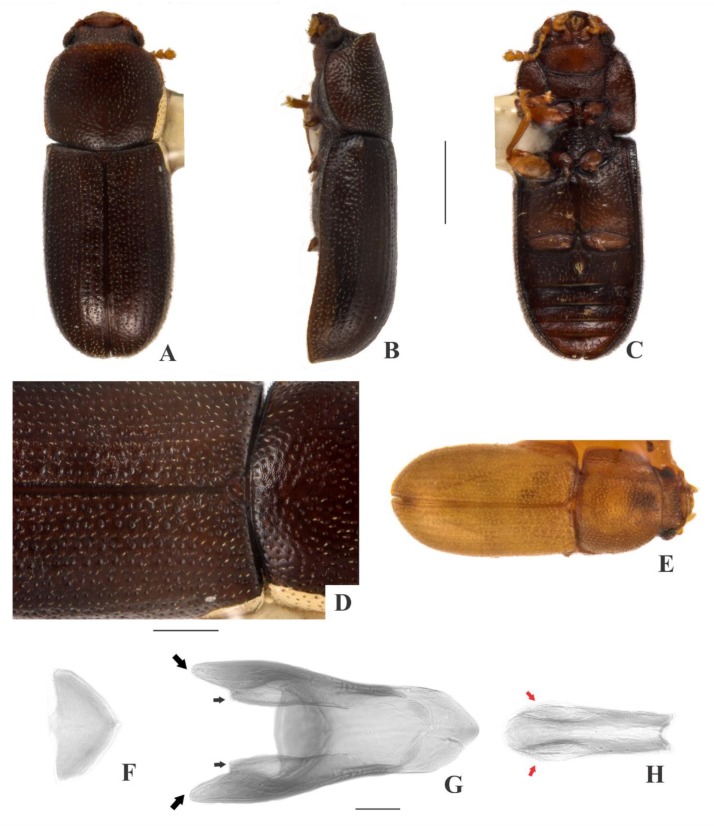
*Cis makrosoma***sp. n.**, male holotype (**A**–**D**), female paratype (**E**), aedeagus of paratype from type locality (**F**–**H**): (**A**) Dorsal view. (**B**) Lateral view. (**C**) Ventral view. (**D**) Scutellar shield and part of the pronotum and elytra. (**F**) Basal piece. (**G**) Tegmen, note acute angulations (big black arrows) with rounded corners (small black arrows) at apex. (**H**) Penis. Scale bars: 0.5 mm (**A**–**C**,**E**), 0.2 mm (**D**), 0.05 mm (**F**–**H**).

**Figure 7:**
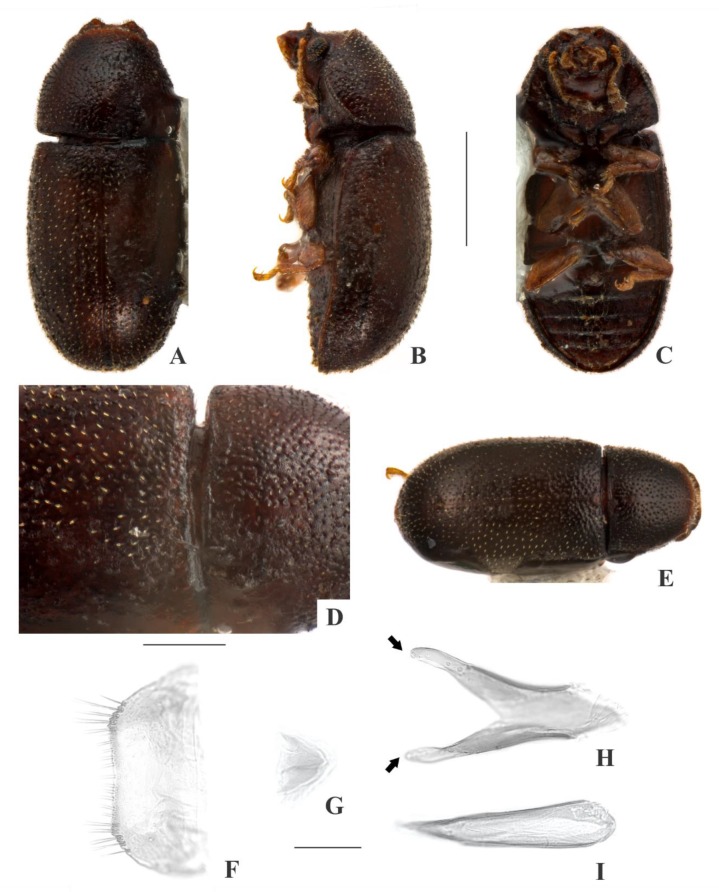
*Cis mpumalangaensis***sp. n.**, male holotype (**A**–**D**), female paratype (**E**), aedeagus of paratype from type locality (**F**–**I**): (**A**) Dorsal view. (**B**) Lateral view. (**C**) Ventral view. (**D**) Scutellar shield and part of the pronotum and elytra. (**F**) Sternite VIII. (**G**) Basal piece. (**H**) Tegmen, note deep V-shaped emargination forming curved slender lateral struts at apex (black arrows). **I.** Penis. Scale bars: 0.5 mm (**A**–**C**, **E**), 0.2 mm (**D**), 0.05 mm (**F**–**I**).

**Figure 8:**
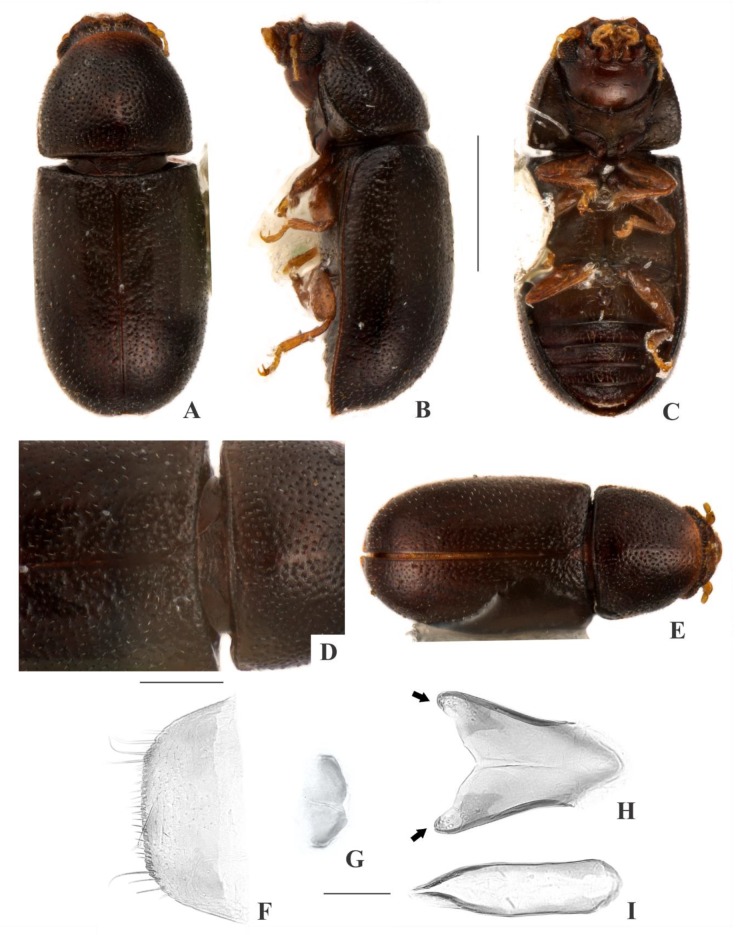
*Cis parvisetosus***sp. n.**, male holotype (**A**–**D**), female paratype (**E**), aedeagus of paratype from type locality (**F**–**I**): (**A**) Dorsal view. (**B**) Lateral view. (**C**) Ventral view. (**D**) Scutellar shield and part of the pronotum and elytra. (**E**) Sternite VIII. (**E**) Basal piece. (**G**) Tegmen, note shallow V-shaped emargination with rounded corners at apex (black arrows). (**H**) Penis. Scale bars: 0.5 mm (**A**–**C**, **E**), 0.2 mm (**D**), 0.05 mm (**F**–**I**).

**Figure 9:**
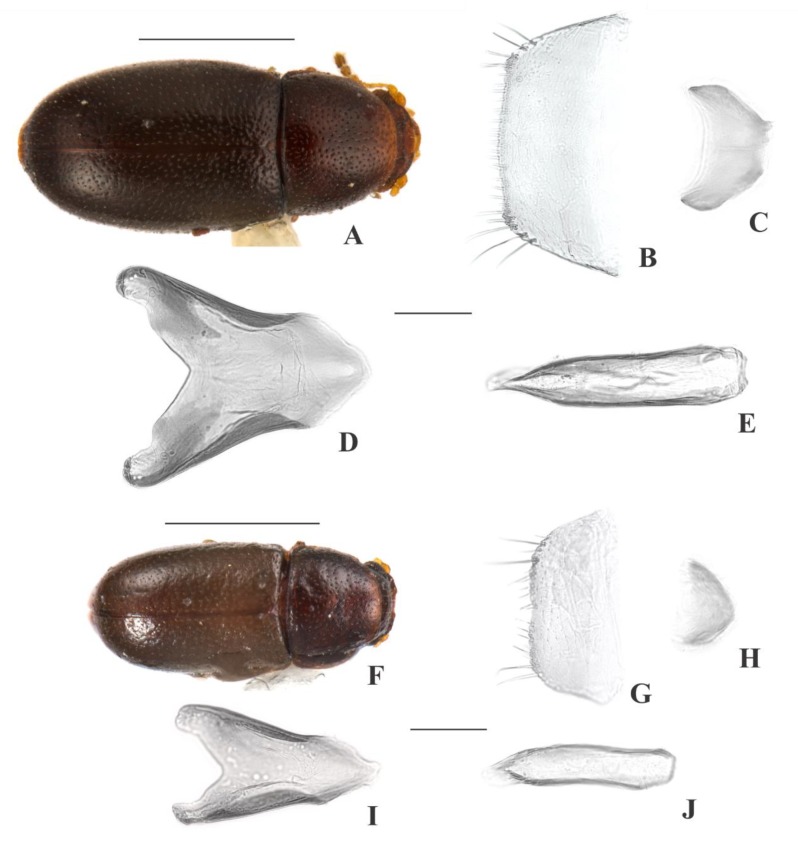
Variation between populations of *Cis parvisetosus* **sp. n.**, male and aedeagus of paratype from Prince Alfred’ S Pass (A–E), male and aedeagus of paratype from Mpisini Nature Reserve (F–J): (**A**,**F**) Dorsal view. (**B**,**G**) Sternite VIII. (**C**,**H**) Basal piece. (**D**,**I**) Tegmen. (**E**,**J**) Penis. Scale bars: 0.5 mm (**A**,**F**), 0.05 mm (**B**–**E**, **G**–**J**).

**Figure 10:**
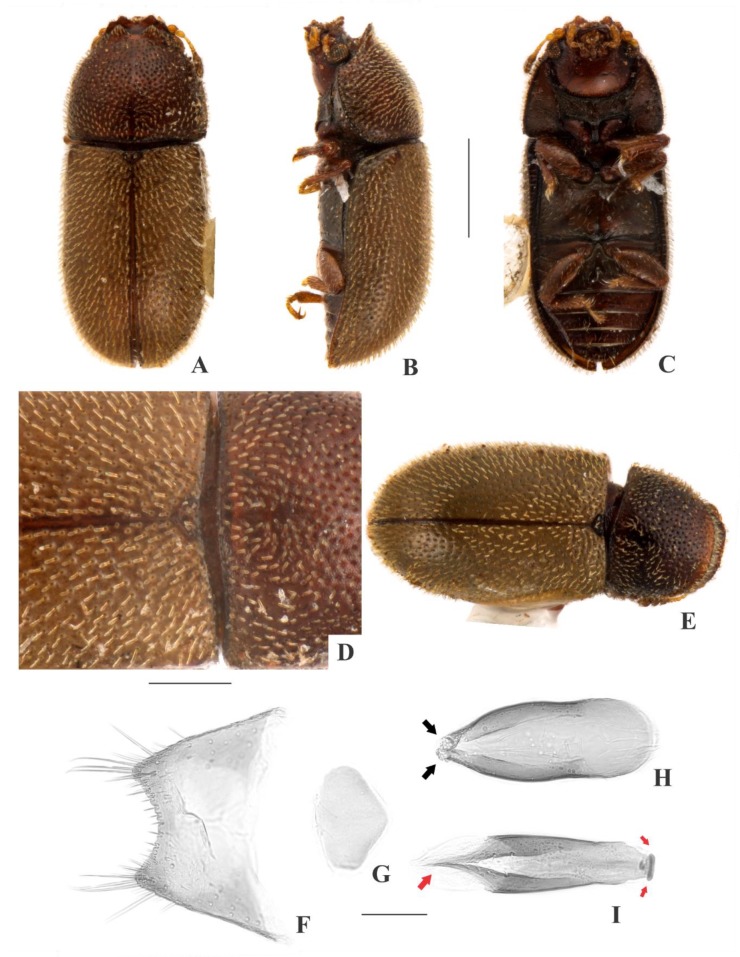
*Cis tessariplacus***sp. n.**, male holotype (**A**–**D**), female paratype (**E**), aedeagus of holotype (**F**–**I**): (**A**) Dorsal view. (**B**) Lateral view. (**C**) Ventral view. (**D**) Scutellar shield and part of the pronotum and elytra. (**F**) Sternite VIII. (**G**) Basal piece. (**H**) Tegmen, note globular tubercles at apical portion (black arrows). (**I**) Penis, note acute sclerotization at middle of apical portion (big red arrow) and truncate sclerotization at anterior portion (small red arrows). Scale bars: 0.5 mm (**A**–**C**,**E**), 0.2 mm (**D**), 0.05 mm (**F**–**I**).

**Figure 11:**
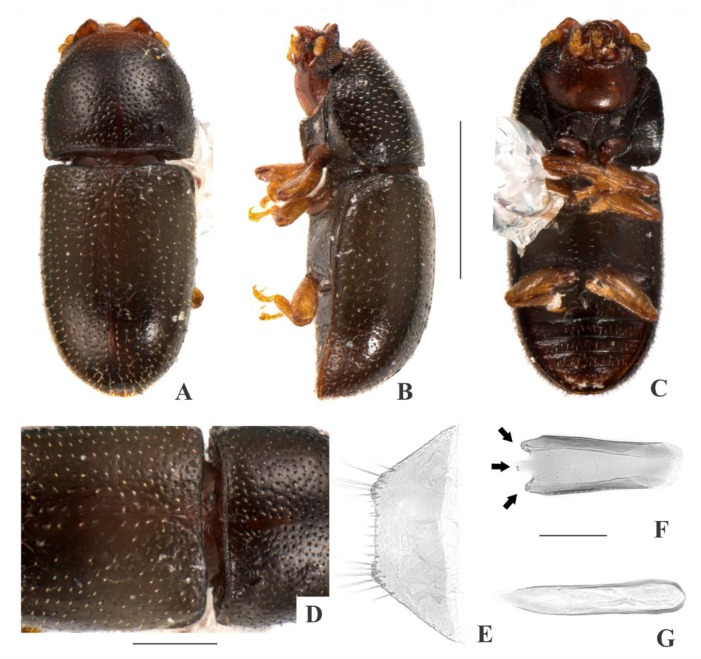
*Cis umlalaziensis***sp. n.**, male holotype (**A**–**D**), aedeagus of paratype from type locality (**E**–**G**): (**A**) Dorsal view. (**B**) Lateral view. (**C**) Ventral view. (**D**) Scutellar shield and part of the pronotum and elytra. (**E**) Sternite VIII. (**F**) Tegmen, note rounded lobes at apex (black arrows). (**G**) Penis. Scale bars: 0.5 mm (**A**–**C**), 0.2 mm (**D**), 0.05 mm (**E**–**G**).

The new species described here (Figures 1–2, Figures 4–11) and part of the new records of previously described species (Figure 3) are based on specimens collected and organized by the staff of the SANC, mostly during 10 years of a project on parasitoids of Ciidae in the Republic of South Africa [9]. The original coding of the morphospecies [9] has been revised and some of them have been described [10,11,12,13]. Correspondence of morphospecies and the new species are cited in the subheadings of each new species.

Terms for external morphology and male terminalia of ciids follow Lawrence [3], Lawrence et al. [14] and Lawrence & Lopes-Andrade [15,16], but see also Oliveira et al. [2] for an explanation on the use of “tegmen”. The following abbreviations are used for measurements (in mm) and ratios: BW (width of anterior edge of scutellar shield), CL (length of antennal club measured from base of the eighth to apex of the tenth antennomere), EL (elytral length along the midline), EW (greatest width of elytra), FL (length of antennal funicle measured from base of the third to apex of the seventh antennomere), GD (greatest depth of body measured in lateral view), GW (greatest diameter of eye), PL (pronotal length along midline), PW (greatest pronotal width), SL (length of scutellar shield), TL (total length counted as EL+PL, i.e., excluding head). The GD/EW and TL/EW ratios indicate the degree of body convexity and elongation, respectively.

Transcription of labels, dissection, photography and measurement of specimens follow the methods provided by Araujo & Lopes-Andrade [17]. Exemplar specimens of the new species from all localities were measured. The number of measured specimens depended on availability and observed variation in size. Differences are given in “Variation”, together with standard measurements (mean and standard deviation) and ratios. Data on host fungi extracted from labels and literature are summarized in the corresponding sections and names were updated consulting the database Index Fungorum [18], together with a corresponding number of records in each fungus species and indicating breeding records. The criteria provided in Orledge and Reynolds [19] was followed for determining breeding records. The distribution maps (Figures 13–15) were created in the freeware QGIS 2.14.2-Essen [20]. Throughout the identification key, provinces of the Republic of South Africa are indicated as EC (Eastern Cape), FS (Free State), GP (Gauteng), KZN (KwaZulu-Natal), LP (Limpopo), MP (Mpumalanga), NW (North West) and WC (Western Cape).

**Figure 12:**
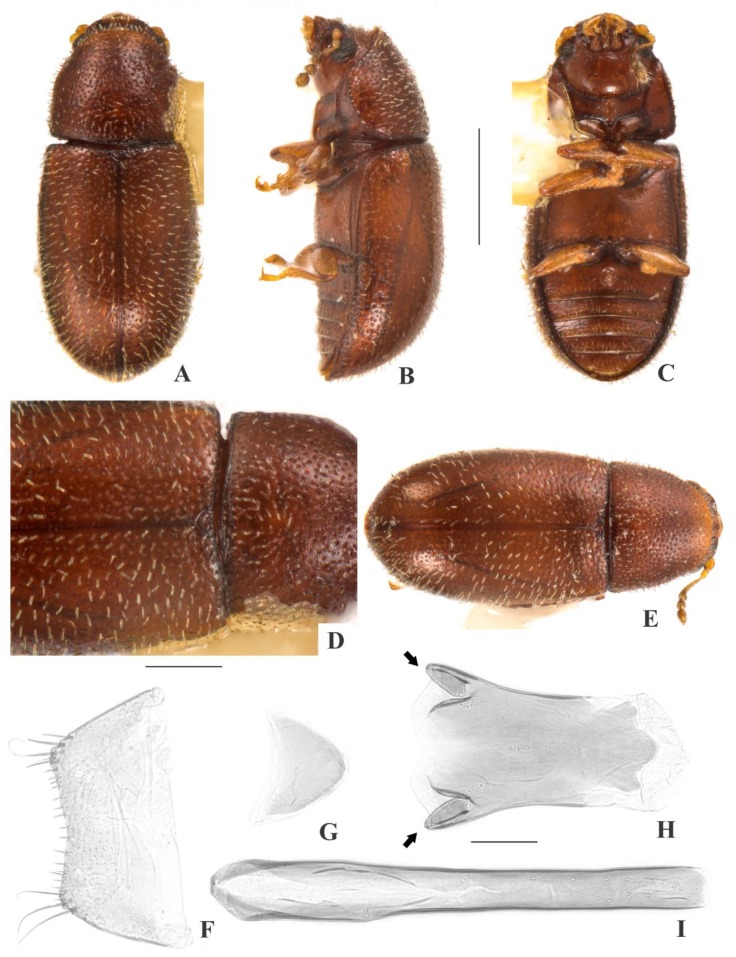
*Cis westerncapensis***sp. n.**, male holotype (**A**–**D**), female paratype (**E**), aedeagus of paratype from Saasveld Forestry College (**F**–**I**): (**A**) Dorsal view. (**B**) Lateral view. (**C**) Ventral view. (**D**) Scutellar shield and part of the pronotum and elytra. (**F**) Sternite VIII. (**G**) Basal piece. (**H**) Tegmen, note acute angulations at apex (black arrows). (**I**) Penis. Scale bars: 0.5 mm (**A**–**C**,**E**), 0.2 mm (**D**), 0.05 mm (**F**–**I**).

The following males were dissected: one *Cis bicaesariatus* **sp. n.** from Die Hel Nature Reserve, Mpumalanga Province; two *Cis foveocephalus* **sp. n.**, from Mooihoek Farm, Mpumalanga Province; four *Cis grobbelaarae* **sp. n.** (three from Die Hel Nature Reserve, Mpumalanga Province; and one from D’Nyala Nature Reserve, Limpopo Province); three *Cis lacinipennis* **sp. n.** (two from Strathdene Farm and one from Cathedral Peaks Forest Station, KwaZulu-Natal Province); one *Cis makrosoma* **sp. n.** from Nelspruit, Mpumalanga Province; two *Cis mpumalangaensis* **sp. n.** from Mooihoek Farm, Mpumalanga Province; three *Cis parvisetosus* **sp. n.** (one from Monk’ S Cowl, one from Mpisini Nature Reserve, KwaZulu-Natal Province; and one from Prince Alfred’ S Pass, Western Cape Province); one *Cis regius* from Storms River Mouth Restcamp, Western Cape Province; two *Cis tessariplacus* **sp. n.** (one from Mooihoek Farm and one from Alkmaar, Mpumalanga Province); one *Cis umlalaziensis* **sp. n.** from Umlalazi Nature Reserve, KwaZulu-Natal Province; two *Cis westerncapensis* **sp. n.** (one from Montagu Pass and one from Saasveld Forestry College, Western Cape Province). The following females were dissected: three *Cis fuscipes* from Garden of Eden Indigenous Forest, Western Cape Province; two *Cis mpumalangaensis* **sp. n.** from Mooihoek Farm, Mpumalanga Province; one *Cis westerncapensis* **sp. n.** from Saasveld Forestry College, Western Cape Province. The sclerites of aedeagi shown in Figures 1(F–I) and 10(F–I) are of the holotypes. The sclerites of aedeagi shown in Figure 10(B–E, G–J) are of paratypes from other localities and demonstrate the low variation between populations. The sclerites of aedeagi shown in Figures 2(F–I), 4(F–I), 5(F–H), 6(F–H), 7(F–I), 8(F–I) and 11(E–G) are of paratypes from type localities. The sclerites of aedeagus shown in Figure 12(F–I) are of a paratype from a locality rather than the type locality, but identified as conspecific to those from the type locality. In the latter case, aedeagus extracted from male of the type locality was a little damaged during dissection and laid in a bad position on the slide. Information on the gula provided in the descriptions is restricted to the ratio of gula width to head width.

The identification key is restricted to species described and examined by us, because we did not have access to any identified material or type specimens of the other southern African species (*C. afer*, *C. bimucronatus*, *C. caffer*, *C. capensis*, *C. delagoensis*, *C. muriceus* and *C. testaceus*). The species are presented in alphabetical order.

## 3. Results

Information on *C. afer*, *C. caffer*, *C. delagoensis*, *C. muriceus* and *C. testaceus* is mostly that from the original descriptions [21,22,23], images of types of identified specimens made available to us (see Acknowledgments) and provided by Ferrer [24]. Information on *C. bimucronatus* and *C. capensis* are from the original descriptions [22,25]. The descriptions of *C. delagoensis* and *C. bimucronatus* are anecdotal; and only the holotype of *C. muriceus* is known, which is a female in poor condition that seems to be teneral (i.e., recently hatched, light-colored adult). Therefore, we have not considered *C. bimocrunatus*, *C. delagoensis* and *C. muriceus* in comparisons provided in the diagnoses.

Among the previously described species of *Cis* from southern Africa, only four of them were placed in species-groups: *C. mooihoekite* and *C. pickeri* (*C. bilamellatus* species-group); and *C. chinensis* and *C. paraliacus* (*C. multidentatus* species-group). The other previously described species were not placed in any group up to date, because it was necessary to describe the other new species at first and compare them to *Cis* from other biogeographic regions [10]. Here we place most described southern African species in species-groups. *Cis muriceus* and *C. tessariplacus* **sp. n.** are the only species that cannot be placed in any previously established group and we prefer not to propose new groups for them.

The following new species-groups are proposed: *C. makrosoma* species-group, *C. neserorum* species-group, *C. regius* species-group and *C. westerncapensis* species-group. The *makrosoma* group is characterized by: (i) anterocephalic edge simple or weakly bidentate; (ii) dual elytral punctation consisting of megapunctures forming more or less regular longitudinal rows, in-between rows filled with micropunctures bearing short bristles; (iii) very elongate, parallel-sided and flattened body; (iv) prosternum moderately long and flat. The *makrosoma* group includes the following species: the southern African *C. makrosoma* **sp. n.**; *C. interpunctatus* Mellié, 1849 from La Reunión, based on examination of material compared with type; *C. cavifrons* Blair, 1940 from Australia, doubtfully included based on the shape of male genitalia [3]. There are other Ethiopian species that may belong to this group, such as *C. mahensis* Scott, 1926 and *C. parallelus* Scott, 1926, but we did not have access to type of material or specimens compared with type. Both *C. mahensis* and *C. parallelus* can be synonyms of *C. interpunctatus* [26].

The *neserorum* group is characterized by: (i) anterocephalic edge and anterior pronotal edge barely to strongly emarginate, forming two short prominences or subtriangular plates in males; (ii) outer apical angle of male protibia projected in a tooth; (iii) dual and non-seriate (confuse) elytral punctation; (iv) pronotal and elytral vestiture indistinctly to distinctly dual. The *neserorum* group includes the following species: the southern African *C. afer*, *C. aster*, *C. bicaesariatus* **sp. n.**, *C. bimucronatus* (doubtfully included), *C. caffer*, *C. delagoensis*, *C. makebae*, *C. mandelai*, *C. masekelai*, *C. neserorum*, *C. stalsi* and *C. testaceus*; and *Cis renominatus* Sandoval-Gómez, Lopes-Andrade & Lawrence, 2014 from Central Africa [27]. The eastern African species *C. eichelbaumi* Reitter, 1908 from Tanzania, *C. pseudosphindus* Reitter, 1908 from Tanzania and Kenya, and *C. usambarinus* Reitter, 1908 from Tanzania may belong to this group, but we not have access to the type or identified material of these species.

The *regius* group is characterized by: (i) head with a peculiar occipital tubercle close to vertex in males; (ii) pronotum with dual punctation, lateral to anterior edges broadly rounded and bearing a row of sparse setae; (iii) elytra with single punctation and vestiture of seriate setae. The unique southern African species included is *C. regius*, but the group also included *C. biacutus* Reitter, 1908 from Tanzania, Madagascar and Seychelles.

The *westerncapensis* group is characterized by: (i) anterocephalic edge and anterior pronotal edge simple; (ii) dual pronotal punctation; (iii) prosternum more or less carinate; (iv) elytral punctation dual and more or less seriate; (v) elytral vestiture single, subseriate to seriate, consisting of moderately short bristles arising from megapunctures. Included species: the southern African *C. lacinipennis* **sp. n.** and *C. westerncapensis* **sp. n.**

Most remaining southern African species are included in previously proposed groups, as follows: *bilamellatus* group (*C. pickeri* and *C. mooihoekite*) (for a definition of the group, see Lawrence [3] and Souza-Gonçalves & Lopes-Andrade [11]); *comptus* group (*C. grobbelaarae* **sp. n.**) (see Lawrence [6] and Kompantsev [28]); *fuscipes* group (*C. capensis* and *C. fuscipes*) (see Lawrence [3]); *multidentatus* group (*C. chinensis* and *C. paraliacus*) (see Lopes-Andrade [7] and Souza-Gonçalves & Lopes-Andrade [12]); and *pacificus* group (*C. foveocephalus* **sp. n.**, *C. mpumalangaensis* **sp. n.**, *C. parvisetosus* **sp. n.** and *C. umlalaziensis* **sp. n.**) (see Lawrence [3]).

### 3.1. Species Accounts

#### 3.1.1. *Cis afer* Fåhraeus, 1871

*Cis afer* Fåhraeus 1871: 671 [21]. Type locality: Caffraria (=Kaffraria), currently Republic of South Africa: Eastern Cape Province (no specific locality); Ferrer 1997: 408 [24] {lectotype designation}.

**Host fungi:** Unknown.

**Distribution:** Ethiopian. Known from Eastern Cape Province (Republic of South Africa).

**Comments:** The species is known only from the type series [21]. There is no further record in the literature, as far as we have traced. This species is a member of the *neserorum* group.

#### 3.1.2. *Cis aster* Souza-Gonçalves et Lopes-Andrade, 2017

*Cis aster* Souza-Gonçalves & Lopes-Andrade 2017: 340 [11]. Type locality: Republic of South Africa: KwaZulu-Natal Province, Twin Streams Nursery Forest.

**Diagnosis:** The species belongs to the *neserorum* group. It differs from other southern African species in the *neserorum* group (except for *C. afer*, *C. bicaesariatus* **sp. n.**, *C. caffer*, *C. makebae* and *C. masekelai*) in males being devoid of concave impression in anterior pronotal portion. *Cis aster* differs from *C. afer* in males with comparatively longer pronotal plates; from *C. bicaesariatus* **sp. n.** in a comparatively longer body and larger prosternal process; and from *C. caffer* in males possessing much closer plates on anterocephalic and anterior pronotal edges. It differs from *C. makebae* and *C. masekelai* in males with first abdominal ventrite devoid of a sex patch.

**Host fungi:***Hexagonia tenuis* (Hook.) Fr. (Polyporaceae), one record and possibly being breeding record [10].

**Distribution.** Ethiopian. Known from Twin Streams Nursery Forest (KwaZulu-Natal Province, Republic of South Africa).

**Comments.** The species is known only from the type series [11]. In the original description, it was mentioned that the species cooccured with the morphospecies Cis sp. Q. However, it was collected alone (corrigendum to Souza-Gonçalves and Lopes-Andrade [11]).

#### 3.1.3. *Cis bicaesariatus* Souza-Gonçalves et Lopes-Andrade, **sp. n.**

ZooBank: http://www.zoobank.org/NomenclaturalActs/83B82A1D-EF13-4253-987F-6BAD2A972CCA

**Figure 13:**
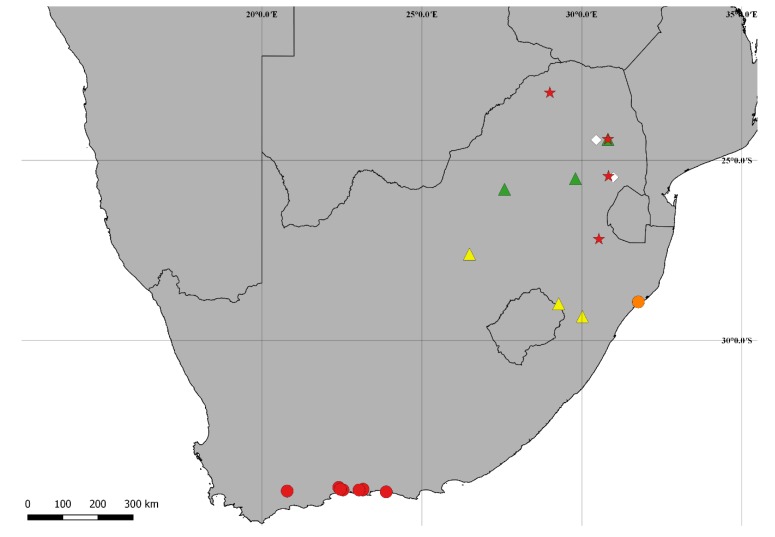
Known distribution of *Cis bicaesariatus* **sp. n.** (green triangle), *Cis lacinipennis* **sp. n.** (yellow triangle), *Cis makrosoma* **sp. n.** (white diamond), *Cis mpumalangaensis* **sp. n.** (red star), *Cis umlalaziensis* **sp. n.** (orange circle) and *Cis westerncapensis* **sp. n.** (red circle).

Figure 1(1–9); Figure 13

*Cis* sp. H in Neser [9].

**Type locality:** “Die Hel Nature Reserve”, coordinates 25°31’ S 29°48’ E (near Loskop Dam, Mpumalanga Province).

**Etymology:** The species name derives from the Latin noun “bi”, which means “two”, and “caesariatus”, which means “covered by hairs”, “long-haired”, both in the genitive singular. The name is a reference to the dual dorsal vestiture of the species.

**Diagnosis:** The species belongs to the *neserorum* group. It differs from other southern African species of the *neserorum* group (except for *C. afer*, *C. aster*, *C. caffer*, *C. makebae* and *C. masekelai*) in males being devoid of concave impression in anterior pronotal portion. *Cis bicaesariatus* **sp. n.** differs from *C. afer*, *C. aster* and *C. caffer* in a comparatively shorter body and in males bearing anterocephalic plates curved upwards and with rounded apex. It differs from *C. makebae* and *C. masekelai* in males with first abdominal ventrite devoid of a sex patch.

**Description, male holotype** (Figure 1(A–D)): Adult apparently not fully pigmented but in good condition, except for lacking three tarsi and for being covered in dust or fungus. Measurements in mm: TL 1.86, PL 0.66, PW 0.81, EL 1.20, EW 0.96, GD 0.71. Ratios: PL/PW 0.82, EL/EW 1.26, EL/PL 1.82, GD/EW 0.74, TL/EW 1.94. ***Body*** elongate, convex, dorsum reddish dark brown (except for dust-covered areas, but visible elsewhere in paratypes); venter reddish dark brown (visible only at abdominal ventrites due to dust-covering, but visible elsewhere in paratypes); antennae yellowish brown with club dark brown, palpi and tarsi yellowish brown; dorsal vestiture distinctly dual, consisting of suberect bristles from distinct lengths, easily discernible in high magnifications (>50×); ventral vestiture consisting of decumbent setae easily discernible in high magnifications (>50×). ***Head*** with anteriormost portion visible from above; dorsum with coarse and deep punctures, separated from each other by less than one puncture-width, with short decumbent bristle (0.01–0.02 mm) in each puncture; interspaces, microreticulate; anterocephalic edge produced and elevated forming two subrounded plates. ***Antennae*** with 10 antennomeres, lengths as follows (in mm, left antennae measured): 0.05, 0.04, 0.03, 0.02, 0.02, 0.02, 0.02, 0.06, 0.07, 0.10 (FL 0.11 mm, CL 0.22 mm, CL/FL 2.05). ***Eyes*** coarsely facetted, with about 90 ommatidia; GW 0.18 mm. ***Gula*** 0.55× as wide as head. ***Pronotum*** (Figure 1(D)) with coarse, deep, single punctation, devoid of impunctate median line; punctures separated from each other by one puncture-width or less; interspaces, microreticulate; vestiture distinctly dual, consisting of moderately long (~0.05 mm) and short (0.02–0.03 mm) suberect yellowish bristles; anterior edge produced and elevated forming two subtriangular plates; lateral edges crenulate, not explanate and not visible when seen from above; anterior corners barely angulate. ***Scutellar shield*** triangular, bearing few punctures and apparently glabrous; BW 0.11 mm; SL 0.09 mm. ***Elytra*** with non-seriate, dual punctation; megapunctures coarse, deep, about 3× as large as micropunctures, separated from each other by one megapuncture-width or less; interspaces a bit rugose; vestiture distinctly dual, consisting of moderately long (0.05–0.06 mm) and short (0.02–0.03 mm) suberect yellowish bristles, both arising from micropunctures. ***Metathoracic wings*** developed, apparently functional. ***Hypomera*** with fine, shallow punctation; each puncture bearing a fine decumbent seta; interspaces, microreticulate. ***Prosternum*** in front of coxae biconcave; interspaces, microreticulate. ***Prosternal process*** parallel-sided, relatively narrow, as long as prosternum at midline, apex truncate. ***Protibiae*** with maximum width about one-third of its length; apical edge devoid of spines; outer apical angle projected in acute tooth. ***Meso- and metatibiae*** without spines in apical edge. ***Metaventrite*** with fine, shallow punctures; interspaces, microreticulate; discrimen about one-third the length of metaventrite at midline. ***Abdominal ventrites*** with fine, shallow punctures, separated from each other by one puncture-width or less and bearing a fine yellowish decumbent seta; interspaces, microreticulate; length of ventrites (in mm, from base to apex at the longitudinal midline) as follows: 0,26, 0.12, 0.10, 0.10, 0.11; first abdominal ventrite devoid of sex patch. ***Male terminalia in a paratype*** (Figure 1(F–I)) with ***sternite VIII*** (Figure 1(F)) with posterior margin rounded, bearing short setae at middle and long at corners; anterio×r portion membranous. ***Tegmen*** (Figure 1(H)) 9.3× as long as wide; widest near apex; subparallel-sided; apex bilobed; apical portion membranous and rounded (Figure 1(H)), black arrows); anterior portion subtriangular. ***Basal piece*** (Figure 1(G)) triangular, as wide as long. ***Penis*** (Figure 1(I)) 1.4× as long as tegmen, 8.9× as long as wide; subparallel-sided; three acute angulations at apex (Figure 1(I), red arrows); shortly rounded emargination at anterior portion.

**Females** (Figure 1(E)): Anterior edge of head barely emarginate and anterior edge of pronotum rounded. Otherwise like males, but without pronotal and head plates and protibial tooth.

**Variation:** Females, measurements in mm (n = 4): TL 1.75–2.00 (1.87 ± 0.12), PL 0.53–0.65 (0.60 ± 0.06), PW 0.73–0.85 (0.79 ± 0.07), EL 1.20–1.35 (1.27 ± 0.07), EW 0.85–0.98 (0.91 ± 0.06), GD 0.65–0.78 (0.72 ± 0.06). Ratios: PL/PW 0.70–0.79 (0.76 ± 0.04), EL/EW 1.37–1.41 (1.39 ± 0.02), EL/PL 2.00–2.33 (2.12 ± 0.14), GD/EW 0.76–0.80 (0.79 ± 0.02), TL/EW 2.00–2.09 (2.05 ± 0.04).

**Type material:** Holotype: ♂ (SANC, dissected) “SOUTH AFRICA: MPU, Die Hel Nature Res., nr. Loskop Dam, 25°31’ S 29°48’ E, 10.viii.2008, S. & O.C. Neser\Ex bracket fungus *Trametes* sp., BF# 147\NATIONAL COLL.OF INSECTS Pretoria, South Africa\*Cis bicaesariatus* Souza-Gonçalves & Lopes-Andrade HOLOTYPUS [red paper]”. Paratypes: 5 ♀♀ as follows: 3 ♀♀ (2 CELC; 1 SANC) same data as the holotype; 1 ♀ (SANC) “SOUTH AFRICA: LIMP, Otter’ S Den 16 Km from Hoedspruit, 24°24’ S 30°49’ E, 18.vii.2008, D. van Heerden\Ex bracket fungus *Coriolus versicolor*, BF# 136\NATIONAL COLL. OF INSECTS Pretoria, South Africa”; 1 ♀ (SANC) “SOUTH AFRICA: NW, Castle Gorge, Magaliesberg, 25°49’ S 27°35’ E, 21.iv.2002, O.C. Neser\Adults emerged from bracket fungus *Phaeolus schweinitzii*, BF# 13\NATIONAL COLL. OF INSECTS Pretoria, South Africa”. All paratypes additionally labeled “*Cis bicaesariatus* Souza-Gonçalves & Lopes-Andrade PARATYPUS [yellow paper]”.

**Host fungi:***Phaeolus schweinitzii* (Fr.) Pat. (Fomitopsidaceae), one record; *Trametes* sp., one record; *Trametes versicolor* (L.) Lloyd (Polyporaceae), one record.

**Distribution:** Ethiopian, known from northern Mpumalanga, southeastern Limpopo and northeastern North West (Republic of South Africa) (Figure 13).

**Comments:** This species was collected together with *C. mandelai*, *C. mpumalangaensis* **sp. n.**, the invasive species *Ceracis tabellifer* (Mellié, 1849) and the parasitoid *Astichus micans* Neser 2012 (Hymenoptera: Eulophidae: Entiinae).

#### 3.1.4. *Cis bimucronatus* Motschoulsky, 1851

*Cis bimucronatus* Motschoulsky 1851: 655 [25]. Type locality: Port Natal, currently Republic of South Africa: KwaZulu-Natal Province, Durban.

**Host fungi:** Unknown.

**Distribution:** Ethiopian. Known from Durban (KwaZulu-Natal Province, Republic of South Africa).

**Comments:** The species is known only from the type series [25]. There is no further record in the literature, as far as we have traced. The description of this species is anecdotal and the type has probably been lost. It is doubtfully included in the *neserorum* group.

#### 3.1.5. *Cis caffer* Fåhraeus, 1871

*Cis caffer* Fåhraeus 1851: 670 [21]. Type locality: Caffraria (=Kaffraria), currently Republic of South Africa: Eastern Cape Province (no specific locality); Ferrer 1997: 408 [24] {lectotype designation}.

**Host fungi:** Unknown.

**Distribution:** Ethiopian. Known from Eastern Cape Province (Republic of South Africa).

**Comments:** The species is known only from the type series [21]. There is no further record in the literature, as far as we have traced. This species is a member of the *neserorum* group.

#### 3.1.6. *Cis capensis* Mellié, 1849

*Cis capensis* Mellié 1849: 254 [22]. Type locality: Republic of South Africa: Western Cape Province, Cape of Good Hope.

**Host fungi:** Unknown.

**Distribution:** Ethiopian. Known from Cape of Good Hope (Western Cape Province, Republic of South Africa).

**Comments:** The species is known only from the type series [22]. There is no further record in the literature, as far as we have traced. This species is a member of the *fuscipes* group and may be even a synonym of *C. fuscipes* properly, a hypothesis that shall be evaluated after careful examination of the type of *C. capensis*.

#### 3.1.7. *Cis chinensis* Lawrence, 1991

*Cis chinensis* Lawrence 1991: 288 [29]. Type locality: China (no specific locality); Madenjian et al. 1993: 47 [30] {found in fungi imported from China to USA, not free-living}; Jinachai et al. 2002 [31] {record from Thailand}; Buder et al. 2008: 171 [4] {GenBank access numbers: FM877940, FM877793, FM877874}; Jelínek 2008: 56 [32] {listed among Palearctic species}; Lopes-Andrade 2008: 36 [7] {record from Brazil}; Rose 2009: 282 [33] {record from France and La Réunion}; Reibnitz & Kunz 2011: 45 [34] {record from Baden-Württemberg, Germany, and Hungary}; Reibnitz 2012 [35] {distribution}; Rose 2012: 346 [36] {host-fungi and record from Launaguet, France}; Diéguez Fernandéz 2013: 104 [37] {record from Spain}; Rose 2014: 1 [38] {record from France}; Amini et al. 2016 [39] {record from Iran}; Lawrence 2016: 42 [3] {redescription and record from Australia}; Rose & Zagatti 2016: 292, 302, 304–305 [40] {host-fungi in France}; Németh et al. 2017: 28 [41] {record from Budapest, Hungary}; Souza-Gonçalves & Lopes-Andrade 2018: 503 [13] {record from Republic of South Africa and Northern Mariana Islands}.

*Cis multidentatus* (Pic 1917) *sensu* Lohse & Reibnitz 1991: 104 [42] {reported from Italy and Germany}; Jelínek 2008: 57 [32] {records from Italy and Germany}; Lopes-Andrade 2008: 42 [7] {reported from Malta}; Shugran et al. 2018: 42 [43] {reported from Iraq}.

*Plesiocis* sp. *sensu* Yan et al. 1998 [44] {record from Shandong province, China}.

**Diagnosis:** The species belongs to the *multidentatus* group. It differs from *C. paraliacus* in bearing elytral punctation single and lateral pronotal edges barely to completely visible from above.

**Host fungi:***Agaricus* sp. (Agaricaceae), one record [42]; *Coprinus* sp. (Agaricaceae), one record [42]; *Daedaleopsis nitida* (Durieu et Mout.) Zmitr. et Malysheva (Polyporaceae), one record [37]; *Fomitopsis pinicola* (Sw.) P. Karst. (Fomitopsidaceae), one record [37]; *Ganoderma lucidum* (Ganodermataceae), seven records as pest of commercial dried fungi [3,29,30,31,32,42]; *Gloeophyllum abietinum* (Bull.) P. Karst. (Gloeophyllaceae), one record [37]; *Lactarius* sp. (Russulaceae), one record [41]; *Pleurotus ostreatus* (Jacq.) P. Kumm. (Pleurotaceae), one record [41]; *Russula* sp. (Russulaceae), one record [41]; *Schizophyllum commune* Fr. (Schizophyllaceae), one breeding record [7]; *Trametes* sp. (Polyporaceae), one breeding record [42]; *Trametes gibbosa* (Pers.) Fr., one record [40].

**Distribution:** Cosmopolitan. Known from east and southeast Asia, northeastern USA, southeastern Brazil, southern, western and eastern Europe, Caucasus, Western Indian Ocean and southern Africa (southern Western Cape, Republic of South Africa). Recorded as pest of the fungus *Ganoderma lucidum* (known as Reishi in Japan and Ling Zhi in China) in east and Southeast Asia [29,30,31] and Australia [3].

**Comments:** This species was collected together with *C. pickeri* and the invasive species *Cer. tabellifer* [13].

#### 3.1.8. *Cis delagoensis* Pic, 1916

*Cis delagoensis* Pic 1916: 14 [23]. Type locality: Mozambique: Delagoa Bay (=Maputo Bay).

**Host fungi:** Unknown.

**Distribution:** Ethiopian. Known from Maputo Bay (Mozambique).

**Comments:** The species is known only from the type series [23]. There is no further record in the literature, as far as we have traced. There are identified specimens in the Natural History Museum (NHM) (pictures examined by us) from Democratic Republic of the Congo, Sierra Leone and Uganda, but we are not sure they are conspecific. This species is a member of the *neserorum* group.

#### 3.1.9. *Cis foveocephalus* Souza-Gonçalves et Lopes-Andrade, **sp. n.**

ZooBank: http://www.zoobank.org/NomenclaturalActs/DD6E566F-C3A5-40BE-8528-582903A57E26

**Figure 14:**
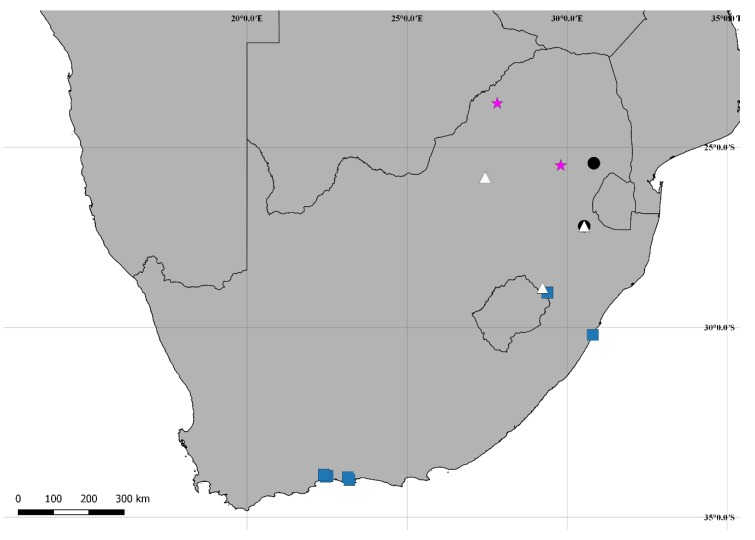
Known distribution of *Cis foveocephalus* **sp. n.** (white triangle), *Cis grobbelaarae* **sp. n.** (pink star), *Cis parvisetosus* **sp. n.** (blue square) and *Cis tessariplacus* **sp. n.** (black circle).

Figure 2(A–I); Figure 14.

*Cis* sp. P in Neser [9].

**Type locality:** “Mooihoek Farm” (near Wakkerstroom), coordinates 27°13’ S 30°32’ E (Pixley Ka Seme Local Municipality, Gert Sibande District, Mpumalanga Province).

**Etymology:** The species name is derived from the Latin noun “foveolae”, which means “small pit”, and the latinized Greek noun “kephale”, which means “head”, both in the genitive singular. The name is a reference to the sex patch present on the head of males.

**Diagnosis:** The species belongs to the *pacificus* group. It differs from all other southern African species of the *pacificus* group in males bearing a sex patch in vertex as well as in first abdominal ventrite.

**Description, male holotype** (Figure 2(A–D)): Adult fully pigmented and in good conditions, except for lacking one leg, five tarsi and both antennae. Measurements in mm: TL 1.75, PL 0.60, PW 0.76, EL 1.15, EW 0.83, GD 0.62. Ratios: PL/PW 0.78, EL/EW 1.39, EL/PL 1.93, GD/EW 0.75, TL/EW 2.11. ***Body*** elongate, convex, dorsum and venter reddish dark brown; palpi and tarsi yellowish brown; dorsal vestiture single, consisting of short suberect bristles, easily discernible in high magnifications (>50×); ventral vestiture of decumbent setae easily discernible in high magnifications (>50×). ***Head*** with anteriormost portion visible from above; dorsum with coarse and deep punctures, separated from each other by one puncture-width or less, with short decumbent bristle (0.01–0.02 mm) arising from each puncture; interspaces, microreticulate; vertex bearing setose sex patch with transverse diameter of 0.06 mm; anterocephalic edge bearing two small triangular tubercles. ***Antennae*** with ten antennomeres, lengths as follows (in mm, left antennae measured in a paratype): 0.05, 0.04, 0.05, 0.04, 0.03, 0.03, 0.02, 0.05, 0.05, 0.06 (FL 0.15 mm, CL 0.15 mm, CL/FL 1.00). ***Eyes*** coarsely facetted, with about 60 ommatidia; GW 0.15 mm. ***Gula*** 0.43× as wide as head. ***Pronotum*** (Figure 2(D)) with irregularly distributed, dual punctation, devoid of impunctate median line; megapunctures coarse, deep, about 2× as large as micropunctures, separated from each other by two megapuncture-widths or less; interspaces, microreticulate; vestiture single, consisting of short suberect yellowish bristles (0.01–0.02 mm) arising from megapunctures; anterior edge rounded; lateral edges crenulate, not explanate and not visible when seen from above; anterior corners rounded. ***Scutellar shield*** triangular, bearing few punctures and few bristles; BW 0.07 mm; SL 0.06 mm. ***Elytra*** with non-seriate, dual punctation; megapunctures coarse, deep, about 2× as large as micropunctures, separated from each other by two megapuncture-widths or less; interspaces a bit rugose; vestiture single, consisting of short suberect yellowish bristles (0.01–0.02 mm) arising from megapunctures. ***Metathoracic wings*** developed, apparently functional. ***Hypomera*** with coarse, shallow punctation; each puncture bearing a fine decumbent seta; interspaces, microreticulate. ***Prosternum*** in front of coxae biconcave and barely carinate; interspaces, microreticulate. ***Prosternal process*** subparallel-sided, about 0.9× as long as prosternum at midline, apex rounded. ***Protibiae*** with maximum width about one-third of its length; apical edge devoid of spines; outer apical angle projected in acute tooth. ***Meso- and metatibiae*** without spines in apical edge. ***Metaventrite*** with coarse, deep punctures; interspaces, microreticulate; discrimen about two-fifths the length of metaventrite at midline. ***Abdominal ventrites*** with coarse, moderately deep punctures, separated from each other by one puncture-width or less and bearing a fine decumbent yellowish seta; interspaces, microreticulate; length of ventrites (in mm, from base to apex at the longitudinal midline) as follows: 0.27, 0.11, 0.09, 0.08, 0.10; first abdominal ventrite bearing margined, circular, setose sex patch posterad of center, with transverse diameter of 0.05 mm. ***Male terminalia in a paratype*** (Figure 2(F–I)) with ***sternite VIII*** (Figure 2(F)) with posterior margin emarginate, bearing short setae at middle and long setae at acute corners; anterior portion membranous. ***Tegmen*** (Figure 2(H)) 3.8× as long as wide, widest near apex; subparallel-sided but more or less sinuate; one rounded emargination in each side forming three lobes at apex, the lateral ones short and acute (Figure 2(H), big black arrows) and the mid one long, somewhat arrow-shaped and covered by sensillae (Figure 2(I), small black arrow); anterior portion triangular. ***Basal piece*** (Figure 2(G)) oval, 1.3× as long as wide. ***Penis*** (Figure 2(I)) 0.9× as long as tegmen, 5.3× as long as wide; subparallel-sided and bearing sensillae near to membranous apex; anterior portion rounded.

**Females** (Figure 2(E)): Anterior edge of head truncate and anterior edge of pronotum rounded. Otherwise like males, but devoid of cephalic tubercles, abdominal and cephalic sex patch, and protibial tooth.

**Variation:** Males, measurements in mm (n = 9, including the holotype): TL 1.43–1.83 (1.65 ± 0.13), PL 0.48–0.65 (0.56 ± 0.06), PW 0.58–0.78 (0.67 ± 0.08), EL 0.95–1.23 (1.09 ± 0.08), EW 0.65–0.88 (0.76 ± 0.07), GD 0.48–0.62 (0.54 ± 0.05). Ratios: PL/PW 0.77–0.96 (0.83 ± 0.07), EL/EW 1.31–1.53 (1.44 ± 0.07), EL/PL 1.77–2.15 (1.58 ± 0.13), GD/EW 0.63–0.77 (0.71 ± 0.04), TL/EW 2.06–2.29 (2.17 ± 0.08). Females, measurements in mm (n = 6): TL 1.30–1.78 (1.61 ± 0.10), PL 0.50–0.55 (0.52 ± 0.02), PW 0.60–0.68 (0.64 ± 0.03), EL 1.00–1.23 (1.10 ± 0.08), EW 0.70–0.78 (0.76 ± 0.03), GD 0.58–0.63 (0.58 ± 0.02). Ratios: PL/PW 0.74–0.88 (0.81 ± 0.05), EL/EW 1.33–1.58 (1.45 ± 0.09), EL/PL 2.00–2.23 (2.12 ± 0.10), GD/EW 0.74–0.82 (0.77 ± 0.04), TL/EW 2.00–2.29 (2.13 ± 0.13).

**Type material:** Holotype: ♂ (SANC) “SOUTH AFRICA: MPU, Mooihoek Farm, nr. Wakkerstroom, 27°13’ S 30°32’ E, 15.vii.2008. O & S Neser\Ex bracket fungus *Stereum ostrea*, BF# 138\NATIONAL COLL. OF INSECTS Pretoria, South Africa\*Cis foveocephalus* Souza-Gonçalves & Lopes-Andrade HOLOTYPUS [red paper]”. Paratypes: 11 ♂♂ and 11 ♀♀ as follows: 6 ♂♂ (2 CELC, dissected; 4 SANC) and 4 ♀♀ (2 CELC; 2 SANC) same data as the holotype; 2 ♂♂ (1 CELC; 1 SANC) and 6 ♀♀ (2 CELC; 4 SANC) “SOUTH AFRICA: MPU, Mooihoek Farm, nr. Wakkerstroom, 27°13’ S 30°32’ E, 15.vii.2008, O & S Neser\Ex bracket fungus, BF# 107\NATIONAL COLL. OF INSECTS Pretoria, South Africa”; 1 ♂ (CELC) “SOUTH AFRICA: NW, Groblerskloof, nr. Buffelspoort Dam, 25°51’ S 27°26’ E, 07.viii.2008, S. Neser\Ex bracket fungus *Trametes cingulata*, BF# 146”; 1 ♂ (SANC) “SOUTH AFRICA: NATAL, Cathedral Peak Forestry Area, 28°55’ S 29°14’ E, 10.xi.1981, SJv Tonder & C Kok \ NATIONAL COLL. OF INSECTS Pretoria, South Africa”; 1 ♂ and 1 ♀ (CELC) “SOUTH AFRICA: NW, Cave Kloof, Magaliesberg, nr. Bufflspoort Dam, 25°51’ S 27°26’ E, 24.vii.2008, S. Neser\Ex bracket fungus, BF# 103\Ex bracket fungus *Ganoderma applanatum*, BF# 103\NATIONAL COLL. OF INSECTS Pretoria, South Africa”. All paratypes additionally labeled “*Cis foveocephalus* Souza-Gonçalves & Lopes-Andrade PARATYPUS [yellow paper]”.

**Host fungi:***Ganoderma applanatum* (Pers.) Pat., one record; *Stereum ostrea* (Blume & T. Nees) Fr., one breeding record; *Tramates cingulata* Berk., one record.

**Distribution:** Ethiopian. Known from southeastern Mpumalanga, northeastern North West and western KwaZulu-Natal (Republic of South Africa) (Figure 14).

**Comments:** This species was collected together with *C. mpumalangaensis* **sp. n.**, *C. tessariplacus* **sp. n.**, the invasive species *Cer. tabellifer* and the parasitoid *A. naiadis* Neser, 2012.

#### 3.1.10. *Cis fuscipes* Mellié, 1849

**Figure 15:**
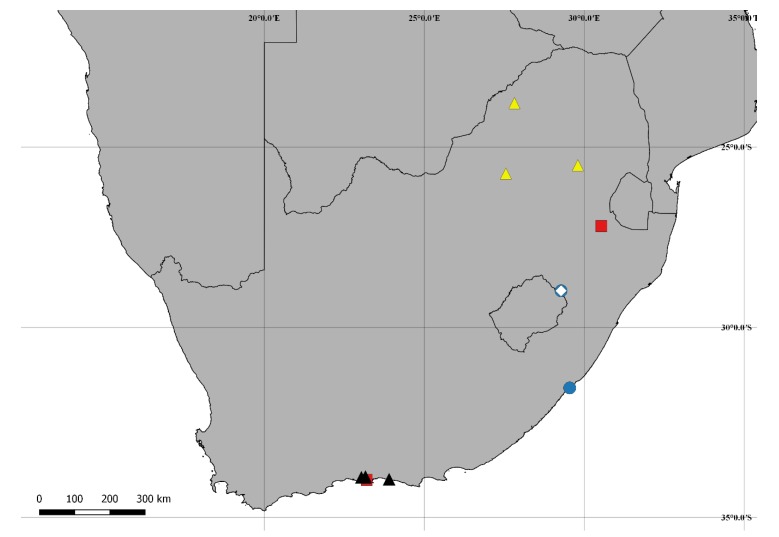
First records of *Cis fuscipes* Mellié (red square), corrected distributional records of *Cis masekelai* Souza-Gonçalves & Lopes-Andrade (yellow triangle) and new distributional records of *Cis neserorum* Souza-Gonçalves & Lopes-Andrade (blue circle), *Cis regius* Orsetti & Lopes-Andrade (black triangle) and *Cis stalsi* Souza-Gonçalves & Lopes-Andrade (white diamond) from southern Africa.

Figure 3(A–D); Figure 15.

*Cis fuscipes* Mellié 1849: 271, pl. 10, Figure 4(A) [22]. Type locality: USA: Massachusetts, Boston; Lawrence 1967: 1–14 [45] {synonyms, distribution and biology}; Lawrence 1971: 460 [6] {distribution and host fungi at North America}; Jelínek 2008: 57 [32] {listed among Paleartic species}; Lawrence 2016: 53 [3] {redescription and record from Australia}; Lopes-Andrade et al. 2016: 347 [46] {record from New Brunswick, Canada}.

*Cis atripennis* Mellié 1849: 258 [22]; Lawrence 1967: 11 [45] {synonym}.

*Cis chevrolatii* Mellié 1849: 249 [22]; Lawrence 1967: 11 [45] {synonym}.

*Cis dubius* Mellié 1849: 273 [22]; Lawrence 1967: 11 [45] {synonym}.

*Cis carolinae* Casey 1898: 78 [47]; Lawrence 1967: 11 [45] {synonym}.

*Cis impressa* Casey 1898: 79 [47]; Lawrence 1967: 11 [45] {synonym}.

*Cis pallens* Casey 1898: 78 [47]; Lawrence 1967: 11 [45] {synonym}.

**Diagnosis:** The species belongs to the *fuscipes* group. It differs from *C. capensis* in males bearing truncate or weakly convex anterocephalic edge and anterior pronotal edge rounded, but only female *C. fuscipes* have been recorded from southern Africa. It differs from females of *C. capensis* in the anteriormost portion of head visible when see from above.

**Additional material:** 6 ♀♀ as follows: 5 ♀♀ (2 CELC, dissected; 3 SANC, one dissected) “SOUTH AFRICA WCape, Garden of Eden Indig. Forest, nr. Knysna, 34°02’ S 23°12’ E, 1.iii.1991, AJ Hendricks\Emerged from log on forest floor of *Olea capensis macrocarpa* OLEACEAE, UA679\NATIONAL COLL. OF INSECTS Pretoria, South Africa”; 1 ♀ (SANC) “SOUTH AFRICA: MPU, Mooihoek Farm, nr. Wakkerstroom, 27°13’ S 30°32’ E, 15.vii.2008, O & S Neser\Ex bracket fungus, BF# 129\Ex bracket fungus *Coriolus versicolor*, BF# 129\NATIONAL COLL. OF INSECTS Pretoria, South Africa”. All additionally labeled “*Cis fuscipes* Mellié, 1849; I. Souza-Gonçalves & C. Lopes-Andrade det.”.

**Host fungi:***Bjerkandera adusta* (Willd.) P. Kasrt. (Merulliaceae), one breeding record [6]; *Cerioporus squamosus* (Huds.) Quél. (Polyporaceae), one breeding record [6,45]; *Daedalea ambigua* Berk. (Fomitopsidaceae), one record [6]; *Fomitopsis pinicola* (Sw.) P. Karst., one record [6]; *Ganoderma applanatum* (Pers.) Pat., one record [6]; *Ganoderma brownii* (Murrill) Gilb. (Ganodermataceae), one breeding record [6,45]; *Lenzites betulinus* (L.) Fr. (Polyporaceae), 12 records, three being breeding records [6]; *Perenniporia fraxinophila* (Peck) Ryvarden, one record [6,45]; *Phellinus gilvus* (Schwein.) Pat. (Hymenochaetaceae), one record [45]; *Poronidulus conchifer* (Schwein.) Murrill (Polyporaceae), three records, two being breeding records [6,45]; *Trametes hirsuta* (Wulfen) Lloyd, 14 records, 10 being breeding records [6,45]; *Trametes pubescens* (Schumach.) Pilát (Polyporaceae), five records, one being breeding record [6]; *Trametes suaveolens* (L.) Fr. (Polyporaceae), one record [45]; *Trametes subectypa* (Murrill) Gilb. & Ryvarden (Polyporaceae), one record [6]; *Trametes versicolor* (L.) Lloyd, 93 records, 48 being breeding records [3,6,45].

**Distribution:** Cosmopolitan. Known from the Holarctic kingdom, widely distributed in North America. Females have been previpously recorded from Australia, Cuba, Hawaii, Madeira and New Zealand, where they were probably introduced [3]. The additional material was collected in southeastern Mpumalanga and southern Western Cape (Republic of South Africa) (Figure 15).

**Comments:** This species was collected together with *C. mooihoekite*, *C. neserorum*, *C. parvisetosus* **sp. n.**, *C. pickeri*, the morphospecies *Cis* sp. S, *Cis* sp. Y and the invasive species *Cer. tabellifer* in South Africa. Only females of *C. fuscipes* were collected by the staff of SANC (Figure 3(A–D)), suggesting that this species may be represented only by parthenogenetic populations in Republic of South Africa.

#### 3.1.11. *Cis grobbelaarae* Souza-Gonçalves et Lopes-Andrade, **sp. n.**

ZooBank: http://www.zoobank.org/NomenclaturalActs/FFEC3023-8443-4DAC-B983-29E0F9C32486

Figure 4(A–I); Figure 14.

*Cis* sp. C in Neser [9].

**Type locality:** “Die Hel Nature Reserve”, coordinates 25°31’ S 29°48’ E (near Loskop Dam, Mpumalanga Province).

**Etymology:** The new species is named in honor of the South African taxonomist Elizabeth Grobbelaar, who collected all paratypes from D’Nyala Nature Reserve. The species name is Latinized from “Grobbelaar” using the feminine suffix in the genitive singular (-*ae*).

**Diagnosis:** The species belongs to the *comptus* group. It differs from all other southern African species (except for *C. makrosoma* **sp. n.**) by the pattern of the dual elytral punctation, consisting of megapunctures forming more or less regular longitudinal rows, in-between rows filled with micropunctures bearing short bristles. It differs from *C. makrosoma* **sp. n.** in males with anterocephalic edge bearing small angulations, less elongated and more convex body, and a comparatively shorter and slightly tumid prosternum.

**Description, male holotype** (Figure 4(A–D)): Adult fully pigmented and in good conditions. Measurements in mm: TL 1.78, PL 0.57, PW 0.71, EL 1.20, EW 0.82, GD 0.62. Ratios: PL/PW 0.80, EL/EW 1.46, EL/PL 2.10, GD/EW 0.75, TL/EW 2.16. ***Body*** elongate, convex, dorsum and venter reddish dark brown; antennae, palpi and tarsi yellowish brown; dorsal vestiture single, consisting of moderately short suberect bristles, easily discernible in low magnifications (<50×); ventral vestiture of decumbent setae easily discernible in low magnifications (<50×). ***Head*** with anteriormost portion visible from above; dorsum with coarse and deep punctures, separated from each other by one puncture-width or less, with short decumbent bristle (0.01–0.02 mm) arising from each puncture; interspaces, microreticulate; anterocephalic edge with four small angulations (barely discernible). ***Antennae*** with 10 antennomeres, lengths as follows (in mm, left antennae measured): 0.07, 0.04, 0.04, 0.03, 0.02, 0.02, 0.02, 0.04, 0.04, 0.06 (FL 0.13 mm, CL 0.15 mm, CL/FL 1.10). ***Eyes*** coarsely facetted, with about 60 ommatidia; GW 0.16 mm. ***Gula*** 0.38× as wide as head. ***Pronotum*** (Figure 4(D)) with coarse, deep, single punctation, devoid of impunctate median line; punctures distributed irregularly, separated from each other by one puncture-width or less; interspaces, microreticulate; vestiture single, consisting of moderately short suberect yellowish bristles (0.03–0.04 mm); anterior edge rounded; lateral edges crenulate, explanate and completely visible when seen from above; anterior corners rounded. ***Scutellar shield*** triangular, bearing few punctures and apparently glabrous; BW 0.14 mm; SL 0.08 mm. ***Elytra*** with seriate, dual punctation; megapunctures coarse, deep, about 2× as large as micropunctures, separated from each other by one megapuncture-width or less, forming more or less longitudinal rows, in-between rows filled with micropunctures; interspaces a bit rugose; vestiture single, consisting of moderately short suberect yellowish bristles (0.03–0.04 mm) arising from micropunctures. ***Metathoracic wings*** developed apparently functional. ***Hypomera*** with coarse, shallow punctation; each puncture bearing a fine decumbent seta; interspaces, microreticulate. ***Prosternum*** in front of coxae slightly tumid; interspaces, microreticulate. ***Prosternal process*** subparallel-sided, as long as prosternum at midline, apex rounded. ***Protibiae*** with maximum width about one-fourth of its length; apical edge devoid of spines; outer apical angle projected in acute tooth. ***Meso- and metatibiae*** without spines in apical edge. ***Metaventrite*** with coarse, deep punctures; interspaces, microreticulate; discrimen about 0.5× the length of metaventrite at midline. ***Abdominal ventrites*** with coarse, moderately deep punctures, separated from each other by one puncture-width or less and bearing a fine decumbent seta; interspaces, microreticulate; length of ventrites (in mm, from base to apex at the longitudinal midline) as follows: 0.24, 0.10, 0.09, 0.09, 0.12; first abdominal ventrite bearing unmargined, small, almost glabrous, sex patch anterad of center, with transverse diameter of 0.04 mm. ***Male terminalia in a paratype*** (Figure 4(F–I)) with ***sternite VIII*** (Figure 4(F)) with the posterior margin barely emarginate, bearing short setae at middle and long setae at rounded corners; anterior portion membranous. ***Tegmen*** (Figure 4(H)) 1.9× as long as wide, widest at apex; sides expanding from basal third to apex; apex with one deep emargination in each side and with acute angulations at corners (Figure 4(H), black arrows); anterior portion rounded. ***Basal piece*** (Figure 4(G)) semicircular, 1.6× as wide as long. ***Penis*** (Figure 4(I)) 0.6× as long as tegmen, 3× as long as wide; sides expanding until basal two-thirds and then converging to apex; apical portion membranous and with sclerotization at middle, apex truncate (Figure 4(I), red arrows); anterior portion with broadly rounded, deep emargination.

**Females** (Figure 4(E)): Anterior edge of head truncate and edge of pronotum rounded. Otherwise like males, but devoid of abdominal sex patch and protibial tooth.

**Variation:** Males, measurements in mm (n = 8, including the holotype): TL 1.38–1.93 (1.73 ± 0.17), PL 0.43–0.63 (0.56 ± 0.06), PW 0.60–0.80 (0.70 ± 0.06), EL 0.95–1.33 (1.18 ± 0.11), EW 0.68–0.88 (0.80 ± 0.07), GD 0.53–0.70 (0.59 ± 0.06). Ratios: PL/PW 0.71–0.86 (0.80 ± 0.06), EL/EW 1.41–1.65 (1.48 ± 0.08), EL/PL 2.00–2.29 (2.12 ± 0.10), GD/EW 0.69–0.90 (0.75 ± 0.07), TL/EW 2.03–2.45 (2.18 ± 0.14). In small males, the angulations at the anterocephalic edge are barely distinguishable. In large males, there are four angulations at the anterocephalic edge (two at corners and two at middle). Females, measurements in mm (n = 7): TL 1.68–2.08 (1.88 ± 0.14), PL 0.53–0.68 (0.60 ± 0.06), PW 0.65–0.85 (0.76 ± 0.07), EL 1.15–1.40 (1.28 ± 0.09), EW 0.75–0.95 (0.88 ± 0.28), GD 0.58–0.75 (0.67 ± 0.06). Ratios: PL/PW 0.74–0.81 (0.79 ± 0.03), EL/EW 1.39–1.53 (1.46 ± 0.06), EL/PL 1.92–2.33 (2.14 ± 0.07), GD/EW 0.72–0.79 (0.76 ± 0.02), TL/EW 2.03–2.23 (2.14 ± 0.07).

**Type material:** Holotype: ♂ (SANC) “SOUTH AFRICA: MPU, Die Hel Nature Res., nr. Loskop Dam, 25°31’ S 29°48’ E, 10.viii.2008, S. & O.C. Neser\Ex unidentified bracket fungus, BF# 122\NATIONAL COLL. OF INSECTS Pretoria, South Africa\*Cis grobbelaarae* Souza-Gonçalves & Lopes-Andrade HOLOTYPUS [red paper]”. Paratypes: 22 ♂♂ and 14 ♀♀ as follows: 4 ♂♂ (1 CELC, dissected; 3 SANC) and 4 ♀♀ (1 CELC; 3 SANC) same data as the holotype; 1 ♂ and 1 ♀ (CELC) “SOUTH AFRICA: MPU, Die Hel Nature Res., nr. Loskop Dam, 25°31’ S 29°48’ E, 10.viii.2008, S. & O.C. Neser\Ex bracket fungus *Trametes meyenii*, BF# 163\NATIONAL COLL. OF INSECTS Pretoria, South Africa”; 7 ♂♂ (2 CELC, one dissected; 5 SANC) and 1 ♀ (SANC) “SOUTH AFRICA: MPU, Die Hel Nature Res., nr. Loskop Dam, 25°31’ S 29°48’ E, 10.viii.2008, S. & O.C. Neser\Ex bracket fungus, BF# 120\Ex bracket fungus *Coriolopsis polyzona*, BF# 120\NATIONAL COLL. OF INSECTS Pretoria, South Africa”; 6 ♂♂ (2 CELC; 4 SANC) and 5 ♀♀ (2 CELC; 3 SANC) “SOUTH AFRICA: MPU, Die Hel Nature Res., nr. Loskop Dam, 25°31’ S 29°48’ E, 10.viii.2008, S. & O.C. Neser\Ex bracket fungus, BF# 119\Ex bracket fungus *Trametes* sp., BF# 119\NATIONAL COLL. OF INSECTS Pretoria, South Africa”; 4 ♂♂ (2 CELC, one dissected; 2 SANC) and 3 ♀♀ (1 CELC; 2 SANC) “SOUTH AFRICA: TvL, D’Nyala Nature Res., near Ellisras, 23.45S 27.49E, 850 m, 29.ix.1989, E. Grobbelaar\Collected from bracket fungus body\NATIONAL COLL. OF INSECTS Pretoria, S. Afr.”. All paratypes additionally labeled “*Cis grobbelaarae* Souza-Gonçalves & Lopes-Andrade PARATYPUS [yellow paper]”.

**Host fungi:***Trametes* sp. (Polyporaceae), one record; *Trametes meyenii* (Klotzsch) Lloyd (Polyporaceae), one record; *Trametes polyzona* (Pers.) Ryvarden (Polyporaceae), one record.

**Distribution:** Ethiopian. Known from northern Mpumalanga and northwestern Limpopo (Republic of South Africa) (Figure 14).

**Comments:** This species was collected together with *C*. *neserorum*, *C. makebae*, *C. masekelai*, *C. mandelai*, *C. mooihoekite*, the morphospecies *Cis* sp. Y, the invasive species *Cer. tabellifer* and the parasitoid *A. micans*.

#### 3.1.12. *Cis lacinipennis* Souza-Gonçalves et Lopes-Andrade, **sp. n.**

ZooBank: http://www.zoobank.org/NomenclaturalActs/36C95C72-DF38-4BAB-A141-216679FA3D58

Figure 5(A–H); Figure 13.

**Type locality:** “Strathedene Farm” (near Nottingham Road), coordinates 29°21’ S 30°01’ E (uMngeni Local Municipality, uMgungundlovu District, KwaZulu-Natal Province).

**Etymology:** The species name derives from the Latin noun “lacina”, which means “flap”, and “penis”, both in the genitive singular. The name is a reference to the shape of the penis of this species, which bears a flap in each side of base.

**Diagnosis:** The species belongs to the *westerncapensis* group. It differs from *C. westerncapensis* **sp. n.** in bearing slightly shorter and seriate vestiture (0.02–0.03 mm), darker dorsal coloration and a comparatively narrower body.

**Description, male holotype** (Figure 5(A–D)): Adult fully pigmented and in good conditions, except for lacking one tarsus. Measurements in mm: TL 1.25, PL 0.40, PW 0.50, EL 0.85, EW 0.56, GD 0.48. Ratios: PL/PW 0.81, EL/EW 1.53, EL/PL 2.11, GD/EW 0.86, TL/EW 2.26. ***Body*** elongate, convex, dorsum and venter reddish dark brown; antennae yellowish brown with club dark brown, palpi and tarsi yellowish brown; dorsal vestiture single, consisting of moderately short suberect bristles, easily discernible in high magnifications (>75×); ventral vestiture of decumbent setae easily discernible in high magnifications (>75×). ***Head*** with anteriormost portion visible from above; dorsum with coarse and deep punctures, separated from each other by one puncture-width or less, with decumbent (~0.02 mm) minute bristle arising from each puncture; interspaces, microreticulate; anterocephalic edge truncate. ***Antennae*** with 10 antennomeres, lengths as follows (in mm, left antennae measured): 0.05, 0.04, 0.03, 0.02, 0.02, 0.02, 0.02, 0.03, 0.03, 0.04 (FL 0.10 mm, CL 0.11 mm, CL/FL 1.07). ***Eyes*** coarsely facetted, with about 50 ommatidia; GW 0.11 mm. ***Gula*** 0.46× as wide as head. ***Pronotum*** (Figure 5(D)) with irregularly distributed, dual punctation bearing an impunctate median line beginning around three punctures-width of base until disc; megapunctures coarse, deep, about 2× as large as micropunctures, separated from each other by one megapuncture-width or less; interspaces, microreticulate; vestiture single, consisting of moderately short suberect yellowish bristles (0.02–0.03 mm) arising from megapunctures; anterior edge rounded; lateral edges barely crenulate, not explanate and not visible when seen from above; anterior corners rounded. ***Scutellar shield*** triangular, bearing few punctures and few bristles; BW 0.07 mm; SL 0.05 mm. ***Elytra*** with subseriate, dual punctation; megapunctures coarse, deep, about 2× as large as micropunctures, separated from each other by two megapuncture-widths or less; interspaces a bit rugose; vestiture seriate, single, consisting of moderately short suberect yellowish bristles (0.02–0.03 mm) arising from megapunctures. ***Metathoracic wings*** developed, apparently functional. ***Hypomera*** with coarse, shallow punctation; each puncture bearing one fine decumbent seta; interspaces, microreticulate. ***Prosternum*** in front of coxae biconcave and barely carinate; interspaces, microreticulate. ***Prosternal process*** subparallel-sided, about 0.9× as long as prosternum at midline, apex rounded. ***Protibiae*** with maximum width about one-fourth of its length; apical edge devoid of spines; outer apical angle projected in acute tooth. ***Meso- and metatibiae*** without spines in apical edge. ***Metaventrite*** with coarse, deep punctures; interspaces, microreticulate; discrimen about one-third the length of metaventrite at midline. ***Abdominal ventrites*** with coarse, moderately deep punctures, separated from each other by one puncture-width or less and bearing one fine decumbent yellowish seta; interspaces, microreticulate; length of ventrites (in mm, from base to apex at the longitudinal midline) as follows: 0.18, 0.07, 0.05, 0.06, 0.08; first abdominal ventrite bearing unmargined, large, circular setose sex patch at middle, with transverse diameter of 0.07 mm. ***Male terminalia in a paratype*** (Figure 5(F–H)) with ***sternite VIII*** (Figure 5(F)) with posterior margin barely emarginate, bearing short setae at middle and long setae at subacute corners; anterior portion membranous. ***Tegmen*** (Figure 5(G)) 1.7× as long as wide, widest at apical third; sides expanding from basal third to apex; apex with rounded emargination and two small tubercles at middle (Figure 5(G), black arrows); anterior portion triangular. ***Penis*** (Figure 5(H)) as long as tegmen, 2.4× as long as wide; subparallel-sided; apex rounded; flaps (Figure 5(H), red arrows) and rounded emargination at anterior portion.

**Females** (Figure 5(36)): Anterior edge of head truncate and anterior edge of pronotum rounded. Otherwise like males, but devoid of abdominal sex patch and protibial tooth.

**Variation:** Males, measurements in mm (n = 12, including the holotype): TL 1.15–1.38 (1.29 ± 0.07), PL 0.38–0.45 (0.41 ± 0.02), PW 0.43–0.53 (0.48 ± 0.04), EL 0.78–0.95 (0.88 ± 0.09), EW 0.50–0.63 (0.58 ± 0.04), GD 0.40–0.53 (0.47 ± 0.04). Ratios: PL/PW 0.80–0.94 (0.85 ± 0.04), EL/EW 1.41–1.71 (1.54 ± 0.11), EL/PL 2.06–2.40 (2.18 ± 0.12), GD/EW 0.73–0.95 (0.82 ± 0.06), TL/EW 2.09–2.50 (2.25 ± 0.14). Females, measurements in mm (n = 11): TL 1.15–1.50 (1.32 ± 0.11), PL 0.38–0.50 (0.42 ± 0.04), PW 0.45–0.65 (0.51 ± 0.06), EL 0.78–1.00 (0.90 ± 0.07), EW 0.55–0.70 (0.61 ± 0.05), GD 0.45–0.58 (0.51 ± 0.04). Ratios: PL/PW 0.75–0.89 (0.83 ± 0.04), EL/EW 1.35–1.59 (1.47 ± 0.07), EL/PL 2.00–2.33 (2.14 ± 0.13), GD/EW 0.78–0.91 (0.84 ± 0.04), TL/EW 2.00–2.27 (2.16 ± 0.08).

**Type material:** Holotype: ♂ (SANC) “SOUTH AFRICA: KZN, Strathdene Farm, nr. Nottingham Rd., 29°21’ S 30°01’ E, 13.vii.2008, S & OC Neser\Ex bracket fungus, BF# 124\NATIONAL COLL. OF INSECTS Pretoria, South Africa\*Cis lacinipennis* Souza-Gonçalves & Lopes-Andrade HOLOTYPUS [red paper]”. Paratypes: 18 ♂♂ and 19 ♀♀ as follows: 8 ♂♂ (3 CELC, 2 dissected; 5 SANC) and 12 ♀♀ (4 CELC; 8 SANC) same data as the holotype; 1 ♂ and 1 ♀ (SANC) “Grootvadersbos, J.K. Grobler, 22.8.1956, Ac.X.846 \ NATIONAL COLL. OF INSECTS Pretoria, S. Afr.”; 5 ♂♂ (1 CELC, dissected; 4 ANIC) and 2 ♀♀ (1 CELC; 1 ANIC) “REP. SOUTH AFRICA: Natal, 75 Km WSW Estcourt Cathedral Peaks For. Sta., 7-31.XII.79, S. & J. Peck \ Ber 28, 29.XII.79, Podocarp forest rotted wood w/fungi, 1500 m”; 2 ♂♂ and 1 ♀ (ANIC) “REP. SOUTH AFRICA: Natal, 75 Km WSW Estcourt Cathedral Peaks For. Sta., 7-31.XII.79, S. & J. Peck\Ber 5, 14.XII.79, Podocarp forest rotted wood, bark, fungi, 1500 m”; 1 ♂ and 1 ♀ (CELC) “REP. SOUTH AFRICA: Natal, 75 Km WSW Estcourt Cathedral Peaks For. Sta., 7-31.XII.79, S. & J. Peck\Ber 6, 15.XII.79, Podocarp forest litter, mossy, boulder bases, 1500m”; 1 ♀ (1 ANIC) “REP. SOUTH AFRICA: Natal, 75 km WSW Estcourt Cathedral Peaks For. Sta., 7-31.XII.79, S. & J. Peck\Ber 1, 10.XII.79, sifted moss, Podocarp forest, 1500m”; 1 ♀ (ANIC) “REP. SOUTH AFRICA: Natal, 75 km WSW Estcourt Cathedral Peaks For. Sta., 7-31.XII.79, S. & J. Peck\Rainbow Gorge, Podocarp For., 1500 m, malaise traps 8, 13.XII.79”. All paratypes additionally labeled “*Cis lacinipennis* Souza-Gonçalves & Lopes-Andrade PARATYPUS [yellow paper]”.

**Host fungi:** Unknown.

**Distribution:** Ethiopian. Known from western KwaZulu-Natal and northern Free State (Republic of South Africa) (Figure 13).

**Comments.** This species was collected together with the invasive species *Cer. tabellifer*.

#### 3.1.13. *Cis makebae* Souza-Gonçalves et Lopes-Andrade, 2017

*Cis makebae* Souza-Gonçalves & Lopes-Andrade 2017: 341 [11]. Type locality: Republic of South Africa: Limpopo Province, Sekororo Kloof.

**Diagnosis:** The species belongs to the *neserorum* group. It differs from other southern African species of the *nesesorum* group (except for *C. afer*, *C. aster*, *C. bicaesariatus* **sp. n.**, *C. caffer* and *C. masekelai*) in males being devoid of concave impression in anterior pronotal portion. *Cis makebae* differs from *C. afer*, *C. aster*, *C. bicaesariatus* **sp. n.** and *C. caffer* in males bearing comparatively shorter anterocephalic and pronotal plates.

**Host fungi:***Trametes cingulata* Berk. (Polyporaceae), two breeding records (Souza-Gonçalves & Lopes-Andrade 2017).

**Distribution.** Ethiopian. Known from northeastern KwaZulu-Natal and southeastern Limpopo (Republic of South Africa).

**Comments:** The species is known only from the type series [11]. This species was collected together with *C. grobbelaarae* **sp. n.**, *C. makrosoma* **sp. n.**, *C. mandelai* and the parasitoid *A. micans* [11].

#### 3.1.14. *Cis makrosoma* Souza-Gonçalves et Lopes-Andrade, **sp. n.**

ZooBank: http://www.zoobank.org/NomenclaturalActs/BD37E9B0-CD68-4768-A7B9-5926792596CA

Figure 6(A–H); Figure 13.

*Cis* sp. G and *Cis* sp. O in Neser [9].

**Type locality:** “Nelspruit”, coordinates 25°29’ S 30°59’ E (Mbombela Municipality, Ehlanzeni District, Mpumalanga Province).

**Etymology:** The species name derives from the Greek adjective “makros”, which means “long”, and the Greek noun “soma”, which means “body”, both in the genitive singular. The name is a reference to the body shape of this species.

**Diagnosis:** The species belongs to the *makrosoma* group. It differs from all other southern African species (except for *C. grobbelaarae* **sp. n.**) by the pattern of the dual elytral punctation, consisting of megapunctures forming more or less regular longitudinal rows, in-between rows filled with micropunctures bearing short bristles. It differs from *C. grobbelaarae* **sp. n.** in males with anterocephalic edge truncate, a very elongated and flattened body, and moderately long and flattened prosternum.

**Description, male holotype** (Figure 6(A–D)): Adult fully pigmented and in good conditions, except for lacking the left antenna and four legs. Measurements in mm: TL 1.88, PL 0.64, PW 0.74, EL 1.24, EW 0.78, GD 0.52. Ratios: PL/PW 0.87, EL/EW 1.59, EL/PL 1.92, GD/EW 0.66, TL/EW 2.41. ***Body*** very elongate, flattened, dorsum and venter reddish dark brown; antennae, palpi and tarsi yellowish brown; dorsal vestiture single, consisting of short suberect bristles, easily discernible in low magnifications (<50×); ventral vestiture of decumbent setae, easily discernible in low magnifications (<50×). ***Head*** with anteriormost portion visible from above; dorsum with coarse and deep punctures, separated from each other by one puncture-width or less, with decumbent minute bristle (0.01–0.02 mm) arising from each puncture; interspaces, microreticulate; anterocephalic edge truncate. ***Antennae*** with 10 antennomeres, lengths as follows (in mm, right antennae measured): 0.05, 0.05, 0.04, 0.03, 0.02, 0.02, 0.02, 0.04, 0.04, 0.05 (FL 0.13 mm, CL 0.12 mm, CL/FL 0.95). ***Eyes*** coarsely facetted, with about 60 ommatidia; GW 0.15 mm. ***Gula*** 0.44× as wide as head. ***Pronotum*** (Figure 6(D)) with coarse, deep, single punctation, bearing an impunctate median line beginning around four puncture-widths of base until disc; punctures distributed irregularly, separated from each other by one to two puncture-widths; interspaces, microreticulate; vestiture single, consisting of suberect yellowish short bristles (~0.01 mm); anterior edge rounded; lateral edges crenulate, barely explanate and completely visible when seen from above; anterior corners barely angulate. ***Scutellar shield*** subtriangular, bearing few punctures and few bristles; BW 0.12 mm; SL 0.06 mm. ***Elytra*** with seriate, dual punctation; megapunctures coarse, deep, about 2× as large as micropunctures, separated from each other by one megapuncture-width or less, forming more or less longitudinal rows, in-between rows filled with micropunctures; interspaces, smooth and shiny; vestiture single, consisting of short suberect yellowish bristles (~0.01 mm) arising from micropunctures. ***Metathoracic wings*** developed, apparently functional. ***Hypomera*** with coarse, shallow punctation; each puncture bearing one fine decumbent seta; interspaces, microreticulate. ***Prosternum*** in front of coxae flattened, moderately long; interspaces, microreticulate. ***Prosternal process*** subparallel-sided, about 0.9× as long as prosternum at midline, apex rounded. ***Protibiae*** with maximum width about one-third of its length; apical edge devoid of spines; outer apical angle projected in acute tooth. ***Meso- and metatibiae*** without spines in apical edge. ***Metaventrite*** with coarse, deep punctures; interspaces, microreticulate; discrimen about one-third the length of metaventrite at midline. ***Abdominal ventrites*** with coarse, moderately deep punctures, separated from each other by one puncture-width or less and bearing one fine decumbent yellowish seta; interspaces, microreticulate; length of ventrites (in mm, from base to apex at the longitudinal midline) as follows: 0.33, 0.11, 0.10. 0.08, 0.11; first abdominal ventrite bearing a margined, large, circular, setose sex patch at center, with transverse diameter of 0.07 mm. ***Male terminalia in a paratype*** (Figure 6(F–H)) with ***sternite VIII*** (laid in bad position during dissection and not shown in figures) with posterior margin almost straight, bearing short setae at middle and long at rounded corners; anterior portion membranous. ***Tegmen*** (Figure 6(G)) 2.0× as long as wide, widest at apical third; sides expanding to apical third; one shallow emargination in each side at apex, forming acute angulation (Figure 6(G), small black arrows) and with rounded corners (Figure 6(G), big black arrows); anterior portion subtriangular. ***Basal piece*** (Figure 6(F)) semicircular, 1.6× as wide as long. ***Penis*** (Figure 6(H)) 0.6× as long as tegmen, 3.0× as long as wide; subparallel-sided; apex rounded and with one excavation in each side (Figure 6(H), red arrows); shortly rounded emargination at anterior portion.

**Females** (Figure 6(E)): Tenerals. Anterior edge of head truncate and anterior edge of pronotum rounded. Otherwise like males, but devoid of abdominal sex patch and protibial tooth.

**Variation:** Males, measurements in mm (n = 4, including the holotype): TL 1.89–1.92 (1.77 ± 0.21), PL 0.49–0.66 (0.59 ± 0.08), PW 0.57–0.74 (0.68 ± 0.08), EL 0.96–1.27 (1.17 ± 0.14), EW 0.68–0.79 (0.75 ± 0.05), GD 0.41–0.54 (0.50 ± 0.06). Ratios: PL/PW 0.86–0.89 (0.87 ± 0.01), EL/EW 1.42–1.66 (1.57 ± 0.10), EL/PL 1.92–2.08 (1.98 ± 0.07), GD/EW 0.61–0.73 (0.67 ± 0.05), TL/EW 2.15–2.46 (2.36 ± 0.15). Females, measurements in mm (n = 3): TL 1.55–1.73 (1.63 ± 0.09), PL 0.53–0.68 (0.59 ± 0.08), PW 0.60–0.65 (0.63 ± 0.03), EL 0.98–1.10 (1.04 ± 0.06), EW 0.65–0.68 (0.67 ± 0.01), GD 0.43–0.48 (0.45 ± 0.03). Ratios: PL/PW 0.81–1.08 (0.95 ± 0.14), EL/EW 1.50–1.63 (1.56 ± 0.07), EL/PL 1.56–2.10 (1.78 ± 0.20), GD/EW 0.65–0.70 (0.67 ± 0.03), TL/EW 2.38–2.56 (2.45 ± 0.09).

**Type material:** Holotype: ♂ (SANC) “SOUTH AFRICA: MPU, Nelspruit, 25°29’ S 30°59’ E, 14.viii.2009, D. van Heerden\Ex bracket fungus, #178, on *Acacia sieberiana* var. *woodii*\Ex bracket fungus *Trametes* sp., BF# 178\NATIONAL COLL. OF INSECTS Pretoria, S. Afr.\*Cis makrosoma* Souza-Gonçalves & Lopes-Andrade HOLOTYPUS [red paper]”. Paratypes: 3 ♂♂ and 3 ♀♀ as follows: 2 ♂♂ (1 CELC, dissected; 1 SANC) same data as the holotype; 1 ♂ (SANC) “SOUTH AFRICA: LIMP, Sekororo Kloof nr. Penge, 24°25’ S 30°27’ E, 27.vii.2008, S. Neser\Ex bracket fungus *Trametes cingulata*, BF# 155\NATIONAL COLL. OF INSECTS Pretoria, South Africa”; 3 ♀♀ (1 CELC; 2 SANC) “N.K. Wildtuin, G.A. Hepburn, 24.3.1960, Ac.X.950\NATIONAL COLL. OF INSECTS Pretoria, S. Afr.”. All paratypes additionally labeled “*Cis makrosoma* Souza-Gonçalves & Lopes-Andrade PARATYPUS [yellow paper]”.

**Host fungi:***Trametes* sp., one record; *Trametes cingulata* Berk. (Polyporaceae), one record.

**Distribution:** Ethiopian. Known from eastern Mpumalanga and southeastern Limpopo (Republic of South Africa) (Figure 13).

**Comments:** This species was collected with *C. makebae*, *C. mandelai*, *C. urbanae*, the invasive species *Cer. tabellifer* and the parasitoid *A. micans*.

#### 3.1.15. *Cis mandelai* Souza-Gonçalves et Lopes-Andrade, 2017

*Cis mandelai* Souza-Gonçalves & Lopes-Andrade 2017: 344 [11]. Type locality: Republic of South Africa: North Western Province, Marethwane.

**Diagnosis:** The species belongs to the *neserorum* group. It differs from other southern African species of the *neserorum* group (except for *C. neserorum*, *C. stalsi*, *C. testaceus*, and *C. urbanae*) in males bearing a concave impression in anterior pronotal portion. *Cis mandelai* differs from *C. neserorum* and *C. stalsi* by its prosternal process, which is conspicuously narrow near the base and gradually expanded to a rounded apex. It differs from *C. urbanae* in the less robust body and males with comparatively shorter pronotal plates; and from *C. testaceus* in bearing head covered by pronotum when seen from above and comparatively shorter pronotal plates with straight sides.

**Host fungi:***Ganoderma* sp. (Ganodermataceae), one breeding record. *Trametes* sp. one breeding record; and *Trametes cingulate* Berk., one breeding record [11].

**Distribution:** Ethiopian. Known from eastern North West, western Gauteng, eastern Mpumalanga and northern Limpopo (Republic of South Africa).

**Comments:** The species is known only from the type series [11]. It was collected together with *C. bicaesariatus* **sp. n.**, *C. grobelaarae* **sp. n.**, *C. makebae*, *C. paraliacus*, *C. parvisetosus* **sp. n.**, *C. neserorum*, *C. westerncapensis* **sp. n.**, the morphospecies *Cis* sp. Y and *Orthocis* sp. A, the invasive species *Cer. tabellifer* and the parasitoid *A. micans* [11].

#### 3.1.16. *Cis masekelai* Souza-Gonçalves et Lopes-Andrade, 2017

*Cis masekelai* Souza-Gonçalves & Lopes-Andrade 2017: 347 [11]. Type locality: Republic of South Africa: Mpumalanga Province, Die Hel Nature Reserve.

**Diagnosis:** The species belongs to the *neserorum* group. It differs from other southern African species of the *neserorum* group (except for *C. afer*, *C. aster*, *C. bicaesariatus* **sp. n.**, *C. caffer* and *C. makebae*) in males being devoid of concave impression in anterior pronotal portion. *Cis masekelai* differs from *C. afer*, *C. aster*, *C. bicaesariatus* **sp. n.**, *C. caffer* and *C. makebae* in males with anterior pronotal edge with a shallow emargination forming two very close short projections.

**Host fungi:***Trametes meyenii* (Klotzsch) Lloyd, two record, one being a breeding record [11].

**Distribution:** Ethiopian. Known from eastern North West, northern Limpopo and eastern Mpumalanga (Republic of South Africa) [11].

**Comments:** The species is known only from the type series [10]. The records provided here (Figure 15) are corrections for that provided by Souza-Gonçalves and Lopes-Andrade [11]. In that paper, the authors cited one recorded as 25°45’ S 27°49’ E instead of 23°45’ S 27°49’ E (*lapsus calami*), here represented by the northernmost record (Figure 15). This species was collected together with *C. neserorum* and the invasive species *Cer. tabellifer.*

#### 3.1.17. *Cis mpumalangaensis* Souza-Gonçalves et Lopes-Andrade, **sp. n.**

ZooBank: http://www.zoobank.org/NomenclaturalActs/80860930-35E0-49E8-8B4E-6167E984E971

Figure 7(A–I); Figure 13.

*Cis* sp. Q and *Cis* sp. N (in part) in Neser [9].

**Type locality:** “Mooihoek Farm” (near Wakkerstroom), coordinates 27°13’ S 30°32’ E (Pixley Ka Seme Local Municipality, Gert Sibande District, Mpumalanga Province).

**Etymology:** The species name is Latinized from “Mpumalanga” in the genitive singular. The name is a reference to the Mpumalanga Province, the province where the holotype and most part of the paratypes were collected.

**Diagnosis:** The species belongs to the *pacificus* group. It differs from *C. foveocephalus* **sp. n.** in males being devoid of a vertexal sex patch; from *C. parvisetosus* **sp. n.** in the comparatively thicker and denser vestiture, as well as longer and more acute anterocephalic plates; and from *C. umlalaziensis* **sp. n.** in the non-subseriate elytral vestiture.

**Description, male holotype** (Figure 7(A–D)): Adult fully pigmented and in good conditions, except for lacking one tarsus. Measurements in mm: TL 1.47, PL 0.49, PW 0.63, EL 0.98, EW 0.69, GD 0.56. Ratios: PL/PW 0.78, EL/EW 1.42, EL/PL 2.01, GD/EW 0.81, TL/EW 2.13. ***Body*** elongate, convex, dorsum and venter reddish dark brown; antennae, palpi and tarsi yellowish brown; dorsal vestiture single, consisting of short suberect bristles, easily discernible in high magnifications (>65×); ventral vestiture of decumbent setae easily discernible in high magnifications (>65×). ***Head*** with anteriormost portion visible from above; dorsum with coarse, deep punctures, separated from each other by less than one puncture-width, with decumbent minute bristles (0.01–0.02 mm) arising from each puncture; interspaces, microreticulate; anterocephalic edge produced and elevated forming two subtriangular plates. ***Antennae*** with 10 antennomeres, lengths as follows (in mm, left antennae measured): 0.05, 0.04, 0.04, 0.03, 0.02, 0.01, 0.01, 0.03, 0.04, 0.05 (FL 0.11 mm, CL 0.12 mm, CL/FL 1.14). ***Eyes*** coarsely facetted; with about 50 ommatidia; GW 0.13 mm. ***Gula*** 0.46× as wide as head. ***Pronotum*** (Figure 7(D)) with irregularly distributed, dual punctation, bearing an impunctate median line beginning about four puncture-widths of base until disc; megapunctures coarse, deep, about 2× as large as micropunctures, separated from each other by one megapuncture-width or less; interspaces, microreticulate; vestiture single, consisting of short suberect yellowish bristles (0.01–0.02 mm) arising from megapunctures; anterior edge rounded; lateral edges not crenulate, not explanate and not visible when seen from above; anterior corners rounded. ***Scutellar shield*** pentagonal, bearing few punctures and few bristles; BW 0.10 mm; SL 0.05 mm. ***Elytra*** with non-seriate, dual punctation; megapunctures coarse, deep, about 2× as large as micropunctures, separated from each other by two puncture-widths or less; interspaces a bit rugose; vestiture single, consisting of short suberect yellowish bristles (0.01–0.02 mm) arising from megapunctures. ***Metathoracic wings*** developed, apparently functional. ***Hypomera*** with coarse, shallow punctation; each puncture bearing one fine decumbent seta; interspaces, microreticulate. ***Prosternum*** in front of coxae biconcave and barely carinate; interspaces, microreticulate. ***Prosternal process*** subparallel-sided, about 0.8× as long as prosternum at midline, apex rounded. ***Protibiae*** with maximum width about one-third of its length; apical edge devoid of spines; outer apical angle projected in acute tooth. ***Meso- and metatibiae*** without spines in apical edge. ***Metaventrite*** with coarse, deep punctures; interspaces, microreticulate; discrimen about one-fourth the length of metaventrite at midline. ***Abdominal ventrites*** with coarse, moderately deep punctures, separated from each other by one puncture-width or less and bearing one fine decumbent yellowish seta; interspaces, microreticulate; length of ventrites (in mm, from base to apex at the longitudinal midline) as follows: 0.22, 0.08, 0.07, 0.07, 0.08; first abdominal ventrite bearing a margined, circular, setose sex patch at middle, with transverse diameter of 0.05 mm. ***Male terminalia in a paratype*** (Figure 7(F–I)) with ***sternite VIII*** (Figure 7(F)) with posterior margin almost straight, bearing short setae at middle and long setae at rounded corners; anterior portion membranous. ***Tegmen*** (Figure 7(H)) 1.9× as long as wide, widest at apex; sides expanding from basal third to apex; apex with deep V-shaped emargination forming slender lateral struts curved to middle (Figure 7(H), black arrows). ***Basal piece*** (Figure 7(G)) subtriangular, 1.4× as wide as long. ***Penis*** (Figure 7(I)) as long as tegmen, 4.6 as long as wide; subcylindrical, subparallel-sided and converging to triangular apex; anterior portion rounded.

**Females** (Figure 7(E)): Anterior edge of head truncate and anterior edge of pronotum rounded. Otherwise like males, but devoid of head plates, abdominal sex patch and protibial tooth.

**Variation:** Males, measurements in mm (n = 5, including the holotype): TL 1.20–1.53 (1.41 ± 0.13), PL 0.40–0.49 (0.47 ± 0.04), PW 0.50–0.63 (0.58 ± 0.05), EL 0.80–1.05 (0.95 ± 0.09), EW 0.60–0.73 (0.67 ± 0.05), GD 0.50–0.56 (0.53 ± 0.02). Ratios: PL/PW 0.78–0.83 (0.81 ± 0.02), EL/EW 1.33–1.46 (1.41 ± 0.05), EL/PL 2.00–2.10 (2.02 ± 0.04), GD/EW 0.76–0.83 (0.80 ± 0.03), TL/EW 2.00–2.19 (2.11 ± 0.07). Females, measurements in mm (n = 8): TL 1.13–1.45 (1.37 ± 0.12), PL 0.38–0.48 (0.43 ± 0.04), PW 0.45–0.63 (0.54 ± 0.06), EL 0.75–1.00 (0.93 ± 0.08), EW 0.55–0.70 (0.64 ± 0.05), GD 0.43–0.55 (0.51 ± 0.04). Ratios: PL/PW 0.75–0.90 (0.81 ± 0.05), EL/EW 1.36–1.56 (1.45 ± 0.06), EL/PL 2.00–2.33 (2.15 ± 0.13), GD/EW 0.68–0.84 (0.80 ± 0.05), TL/EW 2.05–2.28 (2.13 ± 0.08).

**Type material:** Holotype: ♂ (SANC) “SOUTH AFRICA: MPU, Mooihoek Farm, nr. Wakkerstroom, 27°13’ S 30°32’ E, 15.vii.2008, O & S Neser\Ex bracket fungus *Coriolus versicolor*, BF# 135\NATIONAL COLL. OF INSECTS Pretoria, South Africa\*Cis mpumalangaensis* Souza-Gonçalves & Lopes-Andrade HOLOTYPUS [red paper]”. Paratypes: 40 ♂♂ and 32 ♀♀ as follows: 13 ♂♂ (4 CELC, one dissected; 9 SANC) and 15 ♀♀ (5 CELC; 10 SANC) same data as the holotype; 11 ♂♂ (4 CELC, one dissected; 7 SANC) and 5 ♀♀ (3 CELC, one dissected; 2 SANC) “SOUTH AFRICA: MPU, Mooihoek Farm, nr. Wakkerstroom, 27°13’ S 30°32’ E, 15.vii.2008, O & S Neser\Ex bracket fungus *Thelephora* sp., BF# 125\NATIONAL COLL. OF INSECTS Pretoria, South Africa”; 1 ♂ and 2 ♀♀ (CELC) “SOUTH AFRICA: MPU, Mooihoek Farm, nr. Wakkerstroom, 27°13’ S 30°32’ E, 15.vii.2008, O & S Neser\Ex bracket fungus BF# 107\NATIONAL COLL. OF INSECTS Pretoria, South Africa”; 4 ♂♂ (2 CELC; 2 SANC) and 8 ♀♀ (3 CELC; 5 SANC) “SOUTH AFRICA: MPU, Mooihoek Farm, nr. Wakkerstroom, 27°13’ S 30°32’ E, 15.vii.2008, O & S Neser\Ex bracket fungus *Coriolus versicolor*, BF# 140\NATIONAL COLL. OF INSECTS Pretoria, South Africa”; 7 ♂♂ (3 CELC; 4 SANC) and 6 ♀♀ (2 CELC; 4 SANC) “SOUTH AFRICA: MPU, Mooihoek Farm, nr. Wakkerstroom, 27°13’ S 30°32’ E, 15.vii.2008, O & S Neser\Ex bracket fungus *Stereum ostrea*, BF# 138\NATIONAL COLL. OF INSECTS Pretoria, South Africa”; 2 ♂♂ (CELC) “SOUTH AFRICA: MPU, Mooihoek Farm, nr. Wakkerstroom, 27°13’ S 30°32’ E, 15.vii.2008, O & S Neser\Ex bracket fungus *Stereum ostrea*, BF# 90\NATIONAL COLL. OF INSECTS Pretoria, South Africa”; 1 ♂ (SANC) “SOUTH AFRICA: MPU, Mooihoek Farm, nr. Wakkerstroom, 27°13’ S 30°32’ E, 15.vii.2008, O & S Neser\Ex bracket unidentified fungus, BF# 168\NATIONAL COLL. OF INSECTS Pretoria, South Africa”; 2 ♀♀ (CELC) “SOUTH AFRICA: MPU, Mooihoek Farm, nr. Wakkerstroom, 27°13’ S 30°32’ E, 15.vii.2008, O & S Neser\Ex bracket fungus *Trametes* sp., BF# 113\NATIONAL COLL. OF INSECTS Pretoria, South Africa”; 1 ♂ (SANC) “SOUTH AFRICA: MPU, Mooihoek Farm, nr. Wakkerstroom, 27°13’ S 30°32’ E, 15.vii.2008, O & S Neser\Ex bracket fungus *Ganoderma applanatum*, BF# 139\NATIONAL COLL. OF INSECTS Pretoria, South Africa”; 2 ♀♀ (SANC) “SOUTH AFRICA: LIMP, Blouberg Mt. NW Polokwane, 23°04’ S 29°00’ E, 27.iv.2007, OC Neser\Adults ex bracket fungus on fallen trunk\Ex bracket fungus *Trametes* sp., BF# 34\NATIONAL COLL. OF INSECTS Pretoria, South Africa”; 1 ♀ (SANC) “SOUTH AFRICA: LIMP, Otter’ S Dan 16 Km from Hoedspruit, 24°24’ S 30°49’ E, 18.vii.2008, D. van Heerden\Ex bracket fungus *Coriolus versicolor*, BF# 136\NATIONAL COLL. OF INSECTS Pretoria, South Africa”; 1 ♀ (SANC) “SOUTH AFRICA: MPU, Alkmaar W Nelspruit, 25°27’ S 30°50’ E, 10.ii.2008\OC Neser\Ex bracket fungus *Trametes versicolor*, BF# 45\NATIONAL COLL. OF INSECTS Pretoria, South Africa”. All paratypes additionally labeled “*Cis mpumalangaensis* Souza-Gonçalves & Lopes-Andrade PARATYPUS [yellow paper]”.

**Host fungi:***Ganoderma applanatum* (Pers.) Pat., one record; *Stereum ostrea* (Blume & T. Nees) Fr., two records, one being breeding record; *Thelephora* sp. (Thelephoraceae), one breeding record; *Trametes* sp., two records (obs.: we are not sure whether these correspond to a single fungus species or several unidentified *Trametes*); *Trametes versicolor* (L.) Lloyd, four records, two being breeding records.

**Distribution:** Ethiopian. Known from eastern and southeastern Mpumalanga, northern and southeastern Limpopo (Republic of South Africa) (Figure 13).

**Comments:** This species was collected with *C. bicaesariatus* **sp. n.**, *C. foveocephalus* **sp. n.**, *C. neseroum*, *C. mooihoekite*, *C. tessariplacus* **sp. n.**, *Xylographus madagascariensis* Mellié, 1849, Scydmaeninae sp., the invasive species *Cer. tabellifer*, and the parasitoids *A. gracilis* Neser, 2012, *A. micans* and *A. silvani* Neser, 2012.

#### 3.1.18. *Cis mooihoekite* Souza-Gonçalves et Lopes-Andrade, 2018

*Cis mooihoekite* Souza-Gonçalves & Lopes-Andrade 2018: 27 [12]. Type locality: Republic of South Africa: Mpumalanga Province, Mooihoek Farm.

**Diagnosis:** The species belongs to the *bilamellatus* group. It differs from *C. pickeri* in the TL less than 1.30 mm; pronotum devoid of a median impunctate line; anterocephalic edge in male with acute corners and pronotal plate angularly emarginate forming two small and triangular corners with acute apex; and male abdominal sex patch about one-quarter the length of the first ventrite at midline.

**Host fungi:***Thelephora* sp., one record; and *Trametes versicolor* (L.) Lloyd, two records [12].

**Distribution:** Ethiopian. Known from northern and southern Mpumalanga (Republic of South Africa).

**Comments:** The species is known only from the type series [12]. This species was collected together with *C. grobbelaarae* **sp. n.**, *X. madagascariensis*, the invasive species *Cer. tabellifer* and the parasitoids *A. micans* and *A. silvani*.

#### 3.1.19. *Cis muriceus* Mellié, 1849

*Cis muriceus* Mellié 1849: 348 [22]. Type locality: Republic of South Africa: Western Cape Province, Cape of Good Hope.

Host fungi. Unknown.

**Distribution:** Ethiopian. Known from Cape of Good Hope (Western Cape Province, Republic of South Africa).

**Comments:** The species is known only from the type series [22]. There is no further record in the literature, as far as we have traced. We cannot place it in any group at this moment.

#### 3.1.20. *Cis neserorum* Souza-Gonçalves et Lopes-Andrade, 2017

*Cis neserorum* Souza-Gonçalves & Lopes-Andrade 2017: 349 [11]. Type locality: Republic of South Africa: Limpopo Province, Wesfalia Estate.

**Diagnosis:** The species belongs to the *neserorum* group. It differs from other southern African species of the *neserorum* group (except for *C. mandelai*, *C. stalsi*, *C. testaceus*, and *C. urbanae*) in males bearing a concave impression in anterior pronotal portion. *Cis neserorum* differs from *C. mandelai*, *C. stalsi* and *C. urbanae* in males with first abdominal ventrite devoid of a sex patch. It differs from *C. testaceus* in possessing comparatively shorter and closer pronotal plates.

**Additional material:** 3 ♂♂ and 3 ♀♀ as follows: 1 ♂ (ANIC) “REP. SOUTH AFRICA: Natal, 75 Km WSW Estcourt Cathedral Peaks For. Sta., 7-31.XII.74, S. & J. Peck\Ber 28, 29.XII.79, Podocarp forest, rotted wood w/ fungi, 1500m”; 2 ♂♂ and 2 ♀♀ (ANIC) “NATAL, A. JANSE\J.F. Lawrence Lot.2008\ex *Polyporus sanguineus*\ex U.S.D.A. Herbaria”; 1 ♀ (CUIC) “Port St. Johns, U. S. AFR., Mar.6-8.1949, J.C. Bradley\CU [salmon paper]”. All additionally labeled “*Cis neserorum* Souza-Gonçalves & Lopes-Andrade, 2017, I. Souza-Gonçalves det.”

**Host fungi:***Ganoderma applanatum* (Pers.) Pat., one record; *Lenzites elegans* (Spreng.) Pat. (Polyporaceae), six breeding records; *Pycnoporus sanguineus* (L.) Murrill (Polyporaceae), ten records, eight being breeding records; *Stereum ostrea* (Blume & T. Nees) Fr. (Steraceae), one breeding record; *Trametes* sp., six records, three being breeding records (obs.: we are not sure whether these correspond to a single fungus species or several unidentified *Trametes*); *Trametes hirsuta* (Wulfen) Lloyd, four records, three being breeding records; *Trametes meyenii* (Klotzsch) Lloyd, two breeding records; *Trametes polyzona* (Pers.) Justo, four records, three of which are breeding records; *Trametes versicolor* (L.) Lloyd, seven records, six being breeding records; *Thelephora* sp., one breeding record [11].

**Distribution:** Ethiopian. Known from many localities in Republic of South Africa [11]. The species is the largest distributed among *Cis* species in southern and South Africa [11]. The additional material was collected in western and southern KwaZulu-Natal (Republic of South Africa) (Figure 15).

**Comments:** This species was collected together with *C. chinensis*, *C. grobbelaarae* **sp. n.**, *C. fuscipes*, *C. mandelai*, *C. masekelai*, *C. mooihoekite*, *C. mpumalangaensis* **sp. n.**, *C. parvisetosus* **sp. n.**, *C. pickeri*, *C. stalsi*, *C. westerncapensis* **sp. n.**, the morphospecies Cis sp. S, Cis sp. Y, Orthocis sp. A, X. madagascariensis, the invasive species Cer. tabellifer and the parasitoids *A. micans*, *A. gracilis* and *A. silvani*.

#### 3.1.21. *Cis paraliacus* Souza-Gonçalves et Lopes-Andrade, 2018

*Cis paraliacus* Souza-Gonçalves & Lopes-Andrade 2018: 510 [13]. Type locality: Republic of South Africa: KwaZulu-Natal Province, Umlalazi Nature Reserve.

**Diagnosis:** The species belongs to the *multidentatus* group. It differs from *C. chinensis* in bearing elytral punctation dual and lateral protonal edges not visible from above.

**Host fungi:***Trametes hirsuta* (Wulfen) Lloyd, one breeding record [13].

**Distribution:** Ethiopian. Known from eastern and southeastern KwaZulu-Natal (Republic of South Africa).

**Comments:** The species is known only from the type series [13]. This species was collected together with *C. mandelai*, *C. umlalaziensis* **sp. n.**, the invasive species *Cer. tabellifer*, the tenebrionid *Pentaphyllus fronticornis* Gebien, 1910, and the parasitoid *A. micans* [13].

#### 3.1.22. *Cis parvisetosus* Souza-Gonçalves et Lopes-Andrade, **sp. n.**

ZooBank: http://www.zoobank.org/NomenclaturalActs/33ED4C68-7474-4316-BB3C-6734A1067C85

Figure 8(A–I); Figure 9(A–J) Figure 14.

*Cis* sp. M and *Cis* sp. N in Neser [9].

**Type locality:** “Monk’ S Cowl Foothills” (Champagne Castle), coordinates 29°03’ S 29°23’ E (Drakensberg range, KwaZulu-Natal Province).

**Etymology:** The species name derives from the Latin adjectives “parvus”, which means “small”, and “setosum”, which means “setose”, both in the genitive singular. The name is a reference to the short vestiture of this species.

**Diagnosis:** The species belongs to the *pacificus* group. It differs from *C. foveocephalus* **sp. n.** in males being devoid of a vertexal sex patch; from *C. mpumalangaensis* **sp. n.** in the comparatively thinner and sparser vestiture, and comparatively shorter and less acute anterocephalic plates; and from *C. umlalaziensis* **sp. n.** in the non-subseriate and comparatively thinner elytral vestiture.

**Description, male holotype** (Figure 8(A–D)): Adult fully pigmented and in good conditions, except for lacking two tarsi and both antennae. Measurements in mm: TL 1.34, PL 0.46, PW 0.58, EL 0.88, EW 0.65, GD 0.51. Ratios: PL/PW 0.80, EL/EW 1.35, EL/PL 1.89, GD/EW 0.79, TL/EW 2.07. ***Body*** elongate, convex, dorsum and venter reddish dark brown; palpi and tarsi yellowish brown; dorsal vestiture single, consisting of minute suberect setae, easily discernible in high magnifications (>80×); ventral vestiture of decumbent setae easily discernible in high magnifications (>80×). ***Head*** with anteriormost portion visible from above; dorsum with coarse and deep punctures, separated from each other by one puncture-width or less, with suberect minute seta (0.01–0.02 mm) arising from each puncture; interspaces, microreticulate; anterocephalic produced and slightly elevated forming two subtriangular. ***Antennae*** with 10 antennomeres, lengths as follows (in mm, left antennae measured in a paratype): 0.06, 0.05, 0.03, 0.04, 0.03, 0.02, 0.01, 0.03, 0.03, 0.06 (FL 0.13 mm, CL 0.12 mm, CL/FL 0.95). ***Eyes*** coarsely facetted, with about 60 ommatidia; GW 0.13 mm. ***Gula*** 0.55× as wide as head. ***Pronotum*** (Figure 8(D)) with irregularly distributed, dual punctation, bearing an impunctate median line beginning five puncture-widths of base until disc; megapunctures coarse, deep, about 2× as large as micropunctures, separated from each other by one megapuncture-width or less; interspaces, microreticulate; vestiture single, consisting of minute suberect pale yellowish setae (0.01–0.02 mm) arising from megapunctures; anterior edge rounded; lateral edges not crenulate, not explanate and not visible when seen from above; anterior corners rounded. ***Scutellar shield*** triangular, bearing few punctures and apparently glabrous; BW 0.09 mm; SL 0.08 mm. ***Elytra*** with non-seriate, dual punctation; megapunctures coarse, deep, about 2× as large as micropunctures, separated from each other by two megapuncture-widths or less; interspaces a bit rugose; vestiture single, consisting of minute suberect pale yellowish setae (0.01–0.02 mm) arising from megapunctures. ***Metathoracic wings*** developed, apparently functional. ***Hypomera*** with coarse, deep punctation; each puncture bearing a fine decumbent seta; interspaces, microreticulate. ***Prosternum*** in front of coxae biconcave and barely carinate; interspaces, microreticulate. ***Prosternal process*** subparallel-sided, about 0.9× as long as prosternum at midline, apex rounded. ***Protibiae*** with maximum width about one-fourth of its length; apical edge devoid of spines; outer apical angle rounded. ***Meso- and metatibiae*** without spines in apical edge. ***Metaventrite*** with coarse, deep punctures; interspaces, microreticulate; discrimen about one-fifth the length of metaventrite at midline. ***Abdominal ventrites*** with coarse, moderately deep punctures, separated from each other by one puncture-width or less and bearing one fine decumbent pale yellowish seta; interspaces, microreticulate; length of ventrites (in mm, from base to apex at the longitudinal midline) as follows: 0.23, 0.08, 0.08, 0.08, 0.09; first abdominal ventrite bearing margined, circular, setose sex patch at middle, with transverse diameter of 0.05 mm. ***Male terminalia in a paratype*** (Figure 8(F–I)) with ***sternite VIII*** (Figure 8(F)) with posterior margin almost straight, bearing short setae at middle and long setae at rounded corners. ***Tegmen*** (Figure 8(H)) 1.4× as long as wide, widest at apex; sides expanding from basal third to apex; apex with shallow V-shaped emargination; corners rounded (Figure 8(H), black arrows); anterior portion subtriangular. ***Basal piece*** (Figure 8(G)) subhexagonal, 2.8× as long as wide. ***Penis*** (Figure 8(I)) 1.1× as long as tegmen, 4.0× as long as wide; subcylindrical, subparallel-sided and converging to subtriangular apex; anterior portion rounded.

**Females** (Figure 8(E)): Anterior edge of head truncate and anterior edge of pronotum rounded. Otherwise like males, but devoid of abdominal sex patch.

**Variation:** Males, measurements in mm (n = 9, including the holotype): TL 0.91–1.50 (1.26 ± 0.17), PL 0.31–0.50 (0.42 ± 0.06), PW 0.38–0.60 (0.52 ± 0.07), EL 0.60–1.00 (0.84 ± 0.12), EW 0.43–0.70 (0.59 ± 0.08), GD 0.33–0.53 (0.46 ± 0.06). Ratios: PL/PW 0.79–0.90 (0.82 ± 0.03), EL/EW 1.35–1.56 (1.43 ± 0.06), EL/PL 1.84–2.06 (1.98 ± 0.07), GD/EW 0.65–0.90 (0.79 ± 0.07), TL/EW 2.07–2.33 (2.15 ± 0.08). In some populations, the cephalic plates are smaller than than those of males from the type locality (Figure 9(F)) or absent (Figure 9(A)). Some differences are also noted in aedeagus from different populations, principally in size of tegmen and shape of basal piece (Figure 9(B–E,G–J)). Females, measurements in mm (n = 16): TL 0.85–1.53 (1.23 ± 0.17), PL 0.30–0.48 (0.41 ± 0.05), PW 0.30–0.48 (0.41 ± 0.05), EL 0.35–0.58 (0.49 ± 0.06), EW 0.55–1.05 (0.82 ± 0.12), GD 0.40–0.70 (0.57 ± 0.08). Ratios: PL/PW 0.75–0.94 (0.84 ± 0.05), EL/EW 1.31–1.52 (1.43 ± 0.06), EL/PL 1.83–2.27 (2.02 ± 0.11), GD/EW 0.65–0.86 (0.79 ± 0.05), TL/EW 1.96–2.26 (2.15 ± 0.08).

**Type material:** Holotype: ♂ (SANC) “SOUTH AFRICA: KZN, Monk’ S Cowl Foothills Drakensberg, 29°03’ S 29°23’ E, 24.iii.2008, S. & O.C. Neser\Ex bracket fungus on fallen log, BF# 64\Ex bracket fungus *Hymenochaete ochromarginata*, BF# 64\NATIONAL COLL. OF INSECTS Pretoria, South Africa\*Cis parvisetosus* Souza-Gonçalves & Lopes-Andrade HOLOTYPUS [red paper]”. Paratypes: 8 ♂♂ and 20 ♀♀ as follows: 1 ♂ (CELC, dissected) and 2 ♀♀ (1 CELC; 1 SANC) same data as the holotype; 1 ♂ and 2 ♀♀ (SANC) “SOUTH AFRICA: WCAPE, Garden of Eden Indig. Forest, nr. Knysna, 34°02’ S 23°12’ E, 1.iii.1991, AJ Hendricks\Emerged from log of *Nuxia floribunda* BUDDLEJACEAE, UA677B\NATIONAL COLL. OF INSECTS Pretoria, South Africa”; 1 ♂ and 1 ♀ (CELC) “SOUTH AFRICA: WCAPE, Groenkop Indigenous For., near George, 33°56’ S 22°31’ E, 22.iii.1991, AJ Hendricks\Emerged from dead branches of living tree of *Olinia ventosa* OLINIACEAE, UA703\NATIONAL COLL. OF INSECTS Pretoria, South Africa”; 1 ♀ (CELC) “SOUTH AFRICA: WCAPE, Groenkop Indigenous For., near George, 33°56’ S 22°31’ E, 25.iii.1991, AJ Hendricks\Emerged from dead branches of living tree *Rhus chiruadensis* ANACARDIACEAE, UA717\NATIONAL COLL. OF INSECTS Pretoria, South Africa”; 1 ♂ (SANC) “SOUTH AFRICA: WCAPE, Heatherlands, George, 33°57’ S 22°27’ E, 11.iv.1990, AJ Urban\Emerged from *Dais cotinifolia* THYMELAEACEAE, with fruiting body of fungus ?*Lenzites*, UA601\NATIONAL COLL. OF INSECTS Pretoria, South Africa”; 1 ♂ (CELC, dissected) and 4 ♀♀ (2 CELC; 2 SANC) “SOUTH AFRICA: WCAPE, Prince Alfred’ S Pass N of Knysna, 33°58’ S 23°09’ E, 5.xi.2009, S & OC Neser\Ex bracket fungus #215 on fallen tree trunk\Ex bracket fungus *Fomitopsis lilacino-gilva*, BF# 215\NATIONAL COLL. OF INSECTS Pretoria, South Africa”; 1 ♂ and 1 ♀ (CELC) “SOUTH AFRICA WCAPE, Prince Alfred’ S Pass N of Knysna, 33°58’ S 23°09’ E, 5.xi.2009, S & OC Neser\Ex bracket fungus *Coriolus versicolor*, BF# 123\NATIONAL COLL. OF INSECTS Pretoria, South Africa”; 3 ♀♀ (SANC) “SOUTH AFRICA WCAPE, Montagu Pass N of George, 33°54’ S 22°24’ E, 4.xi.2009, S & OC Neser\Ex bracket fungus, #208 on fallen tree trunk\NATIONAL COLL. OF INSECTS Pretoria, South Africa”; 2 ♂♂ (1 CELC, dissected; 1 SANC) and 3 ♀♀ (1 CELC; 2 SANC) “SOUTH AFRICA: KZN, Mpisini Nature Res., 30°12’ S 30°48’ E, 9.vii.2008, S. & O.C. Neser\Ex bracket fungus *Funalia* sp., BF# 110\NATIONAL COLL. OF INSECTS Pretoria, South Africa”; 1 ♀ (SANC) “SOUTH AFRICA: WCAPE, Montagu Pass N of George, 33°54’ S 22°24’ E, 4.xi.2009, S. & O.C. Neser\Ex bracket fungus, #201 on fallen tree trunk\Ex bracket fungus *Ganoderma applanatum*, BF# 201\NATIONAL COLL. OF INSECTS Pretoria, South Africa”; 2 ♀♀ (CELC) “SOUTH AFRICA: WCAPE, Montagu Pass N of George, 33°54’ S 22°24’ E, 4.xi.2009, S. & O.C. Neser\Ex bracket fungus, #210 on fallen tree trunk\Ex bracket fungus *Coriolus hirsutus*, BF# 210\NATIONAL COLL. OF INSECTS Pretoria, South Africa”. All paratypes additionally labeled “*Cis parvisetosus* Souza-Gonçalves & Lopes-Andrade PARATYPUS [yellow paper]”.

**Host fungi:***Funalia* sp. (Polyporaceae), one record; *Ganoderma applanatum* (Pers.) Pat., one record; *Hymenochaete ochromarginata* P.H.B. Talbot (Hymenochaetaceae), one record; *Trametes hirsuta* (Wulfen) Lloyd, one record; *Trametes versicolor* (L.) Lloyd, one record; *Rhodofomitopsis lilacinogilva* (Berk.) B.K. Cui, M.L. Han & Y.C. Dai, one record.

**Distribution:** Ethiopian. Known from southern Western Cape, southeastern and western KwaZulu-Natal (Republic of South Africa) (Figure 14).

**Comments:** This may be a cryptic species and further studies are necessary to elucidate this case. It was collected with *C. fuscipes*, *C. neserorum*, *C. pickeri*, *C. regius*, *C. westerncapensis* **sp. n.**, the morphospecies *Cis* sp. S, *Orthocis* sp. A, Ptinidae sp. 3, *X. madagascariensis*, the invasive species *Cer. tabellifer*, and the parasitoids *A. gracilis* and *A. naiadis*.

#### 3.1.23. *Cis pickeri* Lopes-Andrade, Matushkina, Buder et Klass, 2009

*Cis pickeri* Lopes-Andrade et al. 2009:57 [1]. Type locality: Republic of South Africa: Western Cape Province, Ceres; Souza-Gonçalves & Lopes-Andrade 2018: 31 [12] {new distributional records from Republic of South Africa}.

**Diagnosis:** The species belongs to the *bilamellatus* group. It differs from *C. mooihoekite* in the TL being more than 1.30 mm; pronotum with a median impunctate line; anterocephalic edge in male with rounded corners and pronotal plate slightly emarginate forming two small projections with subrounded apex; and male abdominal sex patch about one-third the length of the first ventrite at midline.

**Host fungi:***Laetiporus* sp. (Fomitopsidaceae), one record; *Russula capensis* A. Person (Russulaceae), one record; and *Trametes versicolor* (L.) Lloyd, one breeding record [12].

**Distribution:** Ethiopian. Known from southern Western Cape (Republic of South Africa).

**Comments:** The species was collected together with *C. bicaesariatus* **sp. n.**, *C. grobbelaarae* **sp. n.**, *C. chinensis*, *C. parvisetosus* **sp. n.**, *C. neserorum*, the morphospecies *Cis* sp. S, *Cis* sp. Y and the invasive species *Cer. tabellifer* [12].

#### 3.1.24. *Cis regius* Orsetti et Lopes-Andrade, 2016

*Cis regius* Orsetti & Lopes-Andrade 2016: 146 [10]. Type locality: Republic of South Africa: Western Cape Province, Prince Alfred’ S Pass.

**Diagnosis:** The species belongs to the *regius* group. It differs from all southern African *Cis* species by the peculiar occipital tubercle close to vertex in males; pronotum with dual punctation, lateral to anterior edges broadly rounded and bearing a row of scattered setae; and elytra with single punctation and vestiture of seriate setae.

**Additional material:** 5 ♂♂ and 4 ♀♀ as follows: 2 ♂♂ (1 CELC, dissected; 1 SANC) and 1 ♀ (SANC) “SOUTH AFRICA: WCAPE, Storms River Mouth, Tsitsikamma National Park, 34°01’ S 23°54’ E, 29.v.2003, S. Neser\Ex bracket fungus [E]\NATIONAL COLL. OF INSECTS Pretoria, South Africa”; 1 ♀ (SANC) “SOUTH AFRICA WCAPE, Diepwalle State Foret, 33°58’ S 23°09’ E, 1.iii.1990, AJ Urban & AJ Hendricks\Collected from seedlings in shaded nursery of *Psoralea pinnata* FABACEAE, UA590:A\NATIONAL COLL. OF INSECTS Pretoria, South Africa”; 1 ♂ and 1 ♀ (ANIC) “REP. S. AFRICA: Cape Prov., Knysna, Diepwalle, 450 m, 17 Dec. 1981, 81–170, forest fungi & litter, S. & J. Peck, coll.”; 2 ♂♂ and 1 ♀ (ANIC) “REP. S. AFRICA: Cape Prov., Gouna, 81–186, 23 Dec. 1981, berlese forest log litter, S. & J. Peck, coll.”. All additionally labeled “*Cis regius* Orsetti & Lopes-Andrade, 2016; I. Souza-Gonçalves det.”.

**Host fungi:***Ganoderma applanatum* (Pers.) Pat., one breeding record [10].

**Distribution:** Ethiopian. Known from southern Western Cape (Republic of South Africa). The additional material was collected in four areas near the type locality (Figure 15).

**Comments:** This species was collected together with *C. parvisetosus* **sp. n.** and the invasive species *Cer. tabellifer*.

#### 3.1.25. *Cis stalsi* Souza-Gonçalves et Lopes-Andrade, 2017

*Cis stalsi* Souza-Gonçalves & Lopes-Andrade 2017: 352 [11]. Type locality: Republic of South Africa: KwaZulu-Natal Province, Hlabisi.

**Diagnosis:** The species belongs to the *neserorum* group. It differs from other southern African species of the *neserorum* group (except for *C. mandelai*, *C. neserorum*, *C. testaceus*, and *C. urbanae*) in males bearing a concave impression in anterior pronotal portion. *Cis stalsi* differs from *C. neserorum* in males with first abdominal ventrite bearing a sex patch. It differs from *C. mandelai* and *C. urbanae* in possessing subparallel-sided prosternal process with rounded apex; and from *C. testaceus* in the comparatively shorter pronotal plates and very close anterocephalic plates.

**Additional material:** 3 ♂♂ and 1 ♀ as follows: 1 ♂♂ and 1 ♀ (ANIC) “REP. SOUTH AFRICA: Natal, 75 Km WSW Estcourt Cathedral Peaks For. Sta., 7-31.XII.79, S. & J. Peck\Ber 22, 26.XII.79, *Eucalyptus* logs, frass & fungi & decaying bark, 1400m”; 2 ♂♂ (ANIC) “REP. SOUTH AFRICA: Natal, 75 km WSW Estcourt Cathedral Peaks For. Sta., 7-31.XII.79, S. & J. Peck\Ber 23, 26.XII.79, *Eucalyptus* logs, decayed litter & soil, 1400 m”. All additionally labeled “*Cis stalsi* Souza-Gonçalves & Lopes-Andrade, 2017; I. Souza-Gonçalves det.”

**Host fungi:***Podoscypha parvula* (Lloyd) D. A. Reid (Meruliaceae), one breeding record [11].

**Distribution:** Ethiopian. Known from eastern North West and eastern KwaZulu-Natal (Republic of South Africa). The additional material was collected in western KwaZulu-Natal (Republic of South Africa) (Figure 15).

**Comments:** This species was collected together with *C. neserorum* and the invasive species *Cer. tabellifer* [11].

#### 3.1.26. *Cis tessariplacus* Souza-Gonçalves et Lopes-Andrade, **sp. n.**

ZooBank: http://www.zoobank.org/NomenclaturalActs/C9186CD7-27CB-4551-BF4B-8E256E3F810C

Figure 10(A–I); Figure 14.

*Cis* sp. L in Neser [9].

**Type locality:** “Mooihoek Farm” (near Wakkerstroom), coordinates 27°13’ S 30°32’ E (Pixley Ka Seme Local Municipality, Gert Sibande District, Mpumalanga Province).

**Etymology:** The species name derives from the Greek noun “tessares”, which means “four”, and “plakos”, which means “plates”, both in the genitive singular. The name is a reference to the number of plates present on the anterocephalic edge of this species.

**Diagnosis:** The species resembles members of the *Cis multidentatus* group in the anterocephalic edge produced and elevate forming four teeth in males and anterior pronotal edge in male projected into two triangular plates; but the prosternum is biconcave and the outer apical angle of protibia is projected in an acute tooth only in males.

**Description, male holotype** (Figure 10(A–D)): Adult apparently not fully pigmented but in good conditions, except for lacking one leg, four tarsi, the left antenna, and for being covered by dust or fungus. Measurements in mm: TL 1.22, PL 0.50, PW 0.75, EL 1.22, EW 0.77, GD 0.59. Ratios: PL/PW 0.67, EL/EW 1.58, EL/PL 2.44, GD/EW 0.77, TL/EW 2.23. ***Body*** elongate, convex, dorsum reddish brown (except for dust-covered areas, but visible elsewhere in paratypes); venter dark reddish brown; antenna yellowish brown with club dark brown, palpi and tarsi yellowish brown; dorsal vestiture dual, consisting of short suberect bristles and minute decumbent setae, easily discernible in high magnifications (>65×); ventral vestiture of decumbent setae easily discernible in high magnifications (>65×). ***Head*** with anteriormost portion visible from above; dorsum with coarse and deep punctures, separated from each other by less than one puncture-width, with one minute decumbent bristle (0.01–0.02 mm) arising from each puncture; interspaces, microreticulate; anterocephalic edge produced and elevated forming four acute and triangular teeth. ***Antennae*** with 10 antennomeres, lengths as follows (in mm, right antennae measured): 0.05, 0.05, 0.05, 0.03, 0.02, 0.02, 0.01, 0.04, 0.04, 0.06 (FL 0.13 mm, CL 0.14 mm, CL/FL 1.07). ***Eyes*** coarsely facetted, with about 60 ommatidia; GW 0.14 mm. ***Gula*** 0.56× as wide as head. ***Pronotum*** (Figure 10(D)) with coarse, deep, single punctation, devoid of an impunctate median line; punctures distributed irregularly, separated from each other by less one puncture-width; interspaces, microreticulate; vestiture single, consisting of short suberect bristles (0.03–0.04 mm); anterior edge produced and elevated forming two triangular plates with acute apex; lateral edges barely crenulate, not explanate and not visible when seen from above; anterior corners rounded. ***Scutellar shield*** pentagonal, bearing few punctures and few bristles; BW 0.13 mm; SL 0.07 mm. ***Elytra*** with non-seriate, single punctation; punctures coarse, deep, distributed irregularly, separated from each other by two puncture-widths or less; interspaces, somewhat a bit rugose; vestiture dual, consisting of short suberect bristles (~0.04 mm) and minute decumbent setae (~0.02 mm), both yellowish and arising from punctures. ***Metathoracic wings*** developed, apparently functional. ***Hypomera*** with coarse, shallow punctation; each puncture bearing one fine decumbent seta; interspaces, microreticulate. ***Prosternum*** in front of coxae biconcave; interspaces, microreticulate. ***Prosternal process*** subparallel-sided, as long as prosternum at midline, apex rounded. ***Protibiae*** with maximum width about one-third of its length; apical edge devoid of spines; outer apical angle projected in acute tooth. ***Meso- and metatibiae*** without spines in apical edge. ***Metaventrite*** with coarse, deep punctures; interspaces, microreticulate; discrimen about two-fifths the length of metaventrite at midline. ***Abdominal ventrites*** with coarse, moderately deep punctures, separated from each other by one puncture-width or less and bearing one fine decumbent yellowish seta; interspaces, microreticulate; length of ventrites (in mm, from base to apex at the longitudinal midline) as follows: 0.24, 0.10, 0.08, 0.07, 0.09; first abdominal ventrite bearing a margined, small, circular, setose sex patch at middle, with transverse diameter of 0.03 mm. ***Male terminalia in a paratype*** (Figure 10(F–I)) with ***sternite VIII*** (Figure 10(F)) with posterior margin deeply emarginate, bearing short setae at middle and long setae at acute corners; anterior margin membranous. ***Tegmen*** (Figure 10(H)) 2.7× as long as wide, widest at middle; small emargination at apex, globular tubercles at apical portion (Figure 10(H), black arrows). ***Basal piece*** (Figure 10(G)) subtrapezoidal, 1.4× as wide as long. ***Penis*** (Figure 10(I)) as long as tegmen, 4.2× as long as wide; sides expanding until apical third and then converging to apex; apical portion membranous and bearing acute sclerotization at middle (Figure 10(I), big red arrow); truncate sclerotization at anterior portion (Figure 10(I), small red arrows).

**Females** (Figure 10(E)): Anterior edge of head truncate and anterior edge of pronotum rounded. Otherwise like males, but devoid of abdominal sex patch and protibial tooth.

**Variation:** Males, measurements in mm (n = 2, including the holotype): TL 1.55–1.72 (1.62 ± 0.14), PL 0.50–0.53 (0.51 ± 0.02), PW 0.60–0.75 (0.67 ± 0.10), EL 1.00–1.22 (1.11 ± 0.16), EW 0.65–0.77 (0.71 ± 0.09), GD 0.48–0.59 (0.53 ± 0.08). Ratios: PL/PW 0.67–0.88 (0.77 ± 0.14), EL/EW 1.54–1.58 (1.56 ± 0.03), EL/PL 1.90–2.44 (2.17 ± 0.38), GD/EW 0.73–0.77 (0.75 ± 0.03), TL/EW 2.23–2.39 (2.29 ± 0.08). Female, measurements in mm (n = 1): TL 1.73, PL 0.58, PW 0.68, EL 1.15, EW 0.83, GD 0.60. Ratios: PL/PW 0.85, EL/EW 1.39, EL/PL 2.00, GD/EW 0.73, TL/EW 2.09.

**Type material:** Holotype: ♂ (SANC, dissected) “SOUTH AFRICA: MPU, Mooihoek Farm, nr. Wakkerstroom, 27°13’ S 30°32’ E, 15.vii.2008, O & S Neser\Ex bracket fungus *Stereum ostrea*, BF# 138\ NATIONAL COLL. OF INSCTS Pretoria, South Africa\*Cis tessariplacus* Souza-Gonçalves & Lopes-Andrade HOLOTYPUS [red paper]”. Paratypes: 1♂ and 1 ♀ as follows: 1♀ (SANC) same data as the holotype; 1♂ (CELC, dissected) “SOUTH AFRICA: MPU, Alkmaar W Nelspruit, 25°27’ S 30°50’ E, 10.ii.2008\OC Neser\Ex bracket fungus *Trametes* sp., BF# 47\NATIONAL COLL. OF INSECTS Pretoria, South Africa”. All paratypes additionally labeled “*Cis tessariplacus* Souza-Gonçalves & Lopes-Andrade PARATYPUS [yellow paper]”.

**Host fungi:***Stereum ostrea* (Blume & T. Nees) Fr., one record; *Trametes* sp., one record.

**Distribution:** Ethiopian. Known from eastern and southeastern Mpumalanga (Republic of South Africa) (Figure 14).

**Comments:** This species was collected together with *C. foveocephalus* **sp. n.**, *C. mpumalangaensis* **sp. n.**, the invasive species *Cer. tabellifer*, and the parasitoids *A. gracilis* and *A. naiadis*. We prefer not to place it in any group at this moment.

#### 3.1.27. *Cis testaceus* Fåhraeus, 1871

*Cis testaceus* Fåhraeus, 1871: 671 [21]. Type locality: Caffraria (=Kaffraria), currently Republic of South Africa: Eastern Cape Province (no specific locality); Ferrer 1997: 408 [24] (lectotype designation).

**Host fungi:** Unknown.

**Distribution:** Ethiopian. Known from Eastern Cape Province (Republic of South Africa).

**Comments:** The species is known only from the type series [21]. There is no further record in the literature, as far as we have traced. It is a member of the *neserorum* group.

#### 3.1.28. *Cis urbanae* Souza-Gonçalves et Lopes-Andrade, 2017

*Cis urbanae* Souza-Gonçalves & Lopes-Andrade 2017: 354 [11]. Type locality: Republic of South Africa: Mpumalanga Province, Nelspruit.

**Diagnosis:** The species belongs to the *neserorum* group. It differs from other southern African species of the *neserorum* group (except for *C. mandelai*, *C. neserorum*, *C. stalsi* and *C. testaceus*) in males bearing a concave impression in anterior pronotal portion. *Cis urbanae* differs from *C. neserorum* in males with first abdominal ventrite bearing a sex patch, and from *C. neserorum* and *C. stalsi* in the prosternal process conspicuously narrow near base and gradually expanding to a rounded apex. *Cis mandelai* bears a similar prosternal process, but differs from in the comparatively more robust body and males with comparatively longer pronotal plates. It differs from *C. testaceus* in the more robust body.

**Host fungi:***Trametes* sp., one breeding record [11].

**Distribution:** Ethiopian. Known from eastern Mpumalanga (Republic of South Africa).

**Comments:** The species is known only from the type series [11]. It was collected together with *C. makrosoma* **sp. n.**, the invasive species *Cer. tabellifer* and the parasitoids *A. micans*.

#### 3.1.29. *Cis umlalaziensis* Souza-Gonçalves et Lopes-Andrade, **sp. n.**

ZooBank: http://www.zoobank.org/NomenclaturalActs/2D0198D9-6915-441E-81A5-FA15FFBCE815

Figure 11(A–G); Figure 13.

*Cis* sp. K in Neser [9].

**Type locality:** “Umlalazi Nature Reserve” (near Mtunzini), coordinates 28°57’ S 31°46’ E (uMlalazi Local Municipality, Uthungulu District, KwaZulu-Natal Province).

**Etymology:** The species name is Latinized from “Umlalazi” in the genitive singular. The name is a reference to the Umlalazi Nature Reserve, the type locality of this species.

**Diagnosis:** The species belongs to the *pacificus* group. It differs from *C. foveocephalus* **sp. n.** in males being devoid of vertexal sex patch; from *C. mpumalangaensis* **sp. n.** in the subseriate elytral vestiture; and from *C. parvisetosus* **sp. n.** in the comparatively thicker and subseriate vestiture, and comparatively longer and more acute anterocephalic plates.

**Description, male holotype** (Figure 11(A–D)): Adult fully pigmented in good conditions, except for lacking both antennae. Measurements in mm: TL 1.11, PL 0.39, PW 0.49, EL 0.72, EW 0.50, GD 0.41. Ratios: PL/PW 0.79, EL/EW 1.45, EL/PL 1.87, GD/EW 0.83, TL/EW 2.22. ***Body*** elongate, convex, dorsum and venter reddish dark brown; palpi and tarsi yellowish brown; dorsal vestiture single, consisting of short suberect bristles, easily discernible in high magnifications (>95×); ventral vestiture of decumbent setae easily discernible in high magnifications (>95×). ***Head*** with anteriormost portion visible from above; dorsum with coarse and deep punctures, separated from each other by one puncture-width or less, with minute decumbent seta (~0.01 mm) arising from each puncture; interspaces, microreticulate; anterocephalic edge produced and elevated forming two triangular plates. ***Antennae*** with 10 antennomeres, lengths as follows (in mm, right antennae measured in a paratype): 0.05, 0.04, 0.03, 0.02, 0.01, 0.01, 0.01, 0.02, 0.03, 0.05 (FL 0.08 mm, CL 0.10 mm, CL/FL 1.25). ***Eyes*** coarsely facetted, with about 50 ommatidia; GW 0.11 mm. ***Gula*** 0.48× as wide as head. ***Pronotum*** (Figure 11(D)) with irregularly distributed, dual punctation, bearing an impunctate median line beginning around four puncture-widths of base until disc; megapunctures coarse, deep, about 2× as large as micropunctures, separated from each other by one puncture-width or less; interspaces, microreticulate; vestiture single, consisting of short suberect yellowish bristles (0.01–0.02 mm) arising from megapunctures; anterior edge rounded; lateral edges not crenulate, not explanate and not visible when seen from above; anterior corners rounded. ***Scutellar shield*** pentagonal, bearing few punctures and few bristles; BW 0.05 mm; SL 0.04 mm. ***Elytra*** with non-seriate, dual punctation; megapunctures coarse, deep, about 2× as large as micropunctures, separated from each other by two puncture-widths or less; interspaces a bit rugose; vestiture subseriate, single, consisting of short suberect yellowish bristles (0.01–0.02 mm) arising from megapunctures. ***Metathoracic wings*** developed, apparently functional. ***Hypomera*** with coarse, shallow punctation; each puncture bearing one fine decumbent seta; interspaces, microreticulate. ***Prosternum*** in front of coxae biconcave and barely carinate; interspaces, microreticulate. ***Prosternal process*** subparallel-sided, about as long as prosternum at midline, apex rounded. ***Protibiae*** with maximum width about one-third of its length; apical edge devoid of spines; outer apical angle projected in acute tooth. ***Meso- and metatibiae*** without spines in apical edge. ***Metaventrite*** with coarse, deep punctures; interspaces, microreticulate; discrimen about one-third the length of metaventrite at midline. ***Abdominal ventrites*** with coarse, moderately deep punctures, separated from each other by one puncture-width or less and bearing one fine decumbent yellowish seta; interspaces, microreticulate; length of ventrites (in mm, from base to apex at the longitudinal midline) as follows: 0.15, 0.06, 0.05, 0.05, 0.07; first abdominal ventrite bearing a unmargined, small, oval, setose sex patch at middle, with transverse diameter of 0.02 mm. ***Male terminalia in a paratype*** (Figure 11(E–G)) with ***sternite VIII*** (Figure 11(E)) with posterior margin almost straight, bearing short setae at middle and long setae at subrounded corners; anterior margin membranous. ***Tegmen*** (Figure 11(F)) 2.8× as long as wide, widest at apical third; sides expanding to apex; apex with one emargination in each side forming three rounded lobes (Figure 11(F), black arrows), the lateral ones slightly shorter than the mid one; anterior portion rounded. ***Penis*** (Figure 11(G)) 1.2× as long as tegmen, 5.8× as long as wide; subcylindrical, subparallel-sided and converging to subtriangular apex; anterior portion rounded.

**Female:** Unknown.

**Variation:** Males, measurements in mm (n = 3, including the holotype): TL 0.98–1.11 (1.05 ± 0.07), PL 0.38–0.39 (0.38 ± 0.01), PW 0.45–0.49 (0.48 ± 0.02), EL 0.60–0.72 (0.68 ± 0.06), EW 0.48–0.50 (0.50 ± 0.01), GD 0.40–0.43 (0.40 ± 0.01). Ratios: PL/PW 0.79–0.83 (0.79 ± 0.03), EL/EW 1.26–1.45 (1.35 ± 0.09), EL/PL 1.60–1.87 (1.80 ± 0.14), GD/EW 0.80–0.89 (0.80 ± 0.05), TL/EW 2.05–2.22 (2.10 ± 0.09).

**Type material:** Holotype: ♂ (SANC) “SOUTH AFRICA: KZN, Umlalazi Nature Res., Mtunzini, 28°57’ S 31°46’ E, 13.vii.2008, R.P. Urban\Ex bracket fungus *Coriolus hirsutus*, BF# 87\NATIONAL COLL. OF INSECTS Pretoria, South Africa\*Cis umlalaziensis* Souza-Gonçalves & Lopes-Andrade HOLOTYPUS [red paper]”. Paratypes: 2 ♂♂ (1 CELC, dissected; 1 SANC) same data as the holotype. All paratypes additionally labeled “*Cis umlalaziensis* Souza-Gonçalves & Lopes-Andrade PARATYPUS [yellow paper]”.

**Host fungi:***Trametes hirsuta* (Wulfen) Lloyd, one record.

**Distribution:** Ethiopian. Known from eastern KwaZulu-Natal (Figure 13).

**Comments:** This species was collected together with *C. paraliacus*, the invasive species *Cer. tabellifer*, the tenebrionid *P. fronticornis* and the parasitoid *A. micans*.

#### 3.1.30. *Cis westerncapensis* Souza-Gonçalves et Lopes-Andrade, **sp. n.**

ZooBank:http://www.zoobank.org/NomenclaturalActs/0DC523DE-0682-4E23-A051-28AC566ADE91

Figure 12(A–I).

*Cis* sp. J in Neser [9].

**Type locality:** “Montagu Pass” coordinates 33°54’ S 22°24’ E (north of George, Western Cape Province).

**Etymology:** The species name is Latinized from “Western Cape” in the genitive singular. The name is a reference to the Western Cape Province, the unique known province where the species occurs.

**Diagnosis:** The species belongs to the *westerncapensis* group. It differs from *C. lacinipennis* **sp. n.** in bearing slightly longer (0.03–0.04 mm) and subseriate vestiture, lighter dorsal coloration and a comparatively wider body.

**Description, male holotype** (Figure 12(A–D)): Fully pigmented and in good conditions, except for lacking the right antenna and one tarsus. Measurements in mm: TL 1.47, PL 0.49, PW 0.58, EL 0.98, EW 0.69, GD 0.55. Ratios: PL/PW 0.84, EL/EW 1.42, EL/PL 2.01, GD/EW 0.80, TL/EW 2.13. ***Body*** elongate, convex, dorsum and venter reddish brown; antenna yellowish brown with club dark brown, palpi and tarsi yellowish brown; dorsal vestiture single, consisting of short suberect bristles, easily discernible in high magnifications (>75×); ventral vestiture of decumbent setae easily discernible in high magnifications (>75×). ***Head*** with anteriormost portion visible from above; dorsum with coarse and deep punctures, separated from each other by one punctures-width or less, with minute decumbent bristle (0.01–0.02 mm) arising from each puncture; interspaces, microreticulate; anterocephalic edge truncate. ***Antennae*** with 10 antennomeres, lengths as follows (in mm, left antenna measured): 0.06, 0.04, 0.03, 0.02, 0.02, 0.02, 0.02, 0.04, 0.04, 0.06 (FL 0.10 mm, CL 0.14 mm, CL/FL 1.42). ***Eyes*** coarsely facetted, with about 50 ommatidia; GW 0.12 mm. ***Gula*** 0.46× as wide as head. ***Pronotum*** (Figure 12(D)) with irregularly distributed, dual punctation, devoid of impunctate median line; megapunctures coarse, deep, about 2× as large as micropunctures, separated from each other by one megapuncture-width or less; interspaces microreticulate; vestiture single, consisting of moderately short suberect pale yellowish bristles (0.02–0.03 mm) arising from micropunctures; anterior edge rounded; lateral edges not crenulate, not explanate and not visible when seen from above; anterior corners rounded. ***Scutellar shield*** pentagonal, bearing few punctures and apparently glabrous; BW 0.08 mm; SL 0.05 mm. ***Elytra*** with subseriate, dual punctation; megapunctures coarse, deep, about 2× as large as micropunctures, separated from each other by two megapuncture-widths or less; interspaces a bit rugose; vestiture subseriate, single, consisting of moderately short suberect pale yellowish bristles (0.03–0.04 mm) arising from megapunctures. ***Metathoracic wings*** developed, apparently functional. ***Hypomera*** with coarse, shallow punctation; each puncture bearing one fine decumbent seta; interspaces, microreticulate. ***Prosternum*** in front of coxae biconcave and barely carinate; interspaces, microreticulate. ***Prosternal process*** subparallel-sided, about 0.9× as long as prosternum at midline, apex rounded. ***Protibiae*** with maximum width about one-fourth of its length; apical edge devoid of spines; outer apical angle projected in acute tooth. ***Meso- and metatibiae*** without spines in apical edge. ***Metaventrite*** with coarse, deep punctures; interspaces, microreticulate; discrimen about one-third the length of metaventrite. ***Abdominal ventrites*** with coarse, moderately deep punctures, separated from each other by one puncture-width or less and bearing one fine decumbent yellowish seta; interspaces, microreticulate; length of ventrites (in mm, from base to apex at the longitudinal midline) as follows: 0.22, 0.08, 0.08, 0.08, 0.10; first abdominal ventrite bearing one margined, large, circular, setose sex patch at middle, with transverse diameter of 0.07 mm. ***Male terminalia in a paratype*** (Figure 12(F–I)) with ***sternite VIII*** (Figure 12(F)) with posterior margin almost straight, bearing short setae at middle and long setae at rounded corners; anterior portion membranous. ***Tegmen*** (Figure 12(H)) 1.8× as long as wide, widest at apex; subparallel-sided; two acute angulations in each side at apex (Figure 12(H), black arrows); anterior portion subrounded. ***Basal piece*** (Figure 12(G)) subtriangular, 1.4× as wide as long. ***Penis*** (Figure 12(I)) 4.3× as long as tegmen, 10.0× as long as wide; subcylindrical, subparallel-sided, somewhat stick-shaped; anterior and posterior portions rounded.

**Females** (Figure 12(E)): Anterior edge of head truncate and anterior edge of pronotum rounded. Otherwise like males, but devoid of abdominal sex patch and protibial tooth.

**Variation:** Males, measurements in mm (n = 21, including the holotype): TL 1.23–1.65 (1.35 ± 0.10), PL 0.43–0.58 (0.46 ± 0.04), PW 0.48–0.65 (0.33 ± 0.05), EL 0.80–1.08 (0.90 ± 0.07), EW 0.55–0.80 (0.63 ± 0.06), GD 0.45–0.63 (0.51 ± 0.04). Ratios: PL/PW 0.77–0.95 (0.86 ± 0.05), EL/EW 1.33–1.58 (1.43 ± 0.70), EL/PL 1.75–2.24 (1.97 ± 0.13), GD/EW 0.78–0.88 (0.81 ± 0.02), TL/EW 2.04–2.33 (2.16 ± 0.09). Females, measurements in mm (n = 28): TL 1.23–1.58 (1.36 ± 0.09), PL 0.40–0.53 (0.45 ± 0.04), PW 0.45–0.58 (0.51 ± 0.03), EL 0.83–1.05 (0.91 ± 0.06), EW 0.53–0.70 (0.62 ± 0.04), GD 0.45–0.58 (0.51 ± 0.04). Ratios: PL/PW 0.81–1.00 (0.89 ± 0.05), EL/EW 1.36–1.58 (1.46 ± 0.06), EL/PL 1.81–2.24 (2.00 ± 0.12), GD/EW 0.76–0.90 (0.83 ± 0.04), TL/EW 2.07–2.38 (2.20 ± 0.08).

**Type material:** Holotype: ♂ (SANC) “SOUTH AFRICA WCAPE, Montagu Pass N of George, 33°54’ S 22°24’ E, 4.xi.2009, S & OC Neser\Ex bracket fungus, #210 on fallen tree trunk\Ex bracket fungus *Coriolus hirsutus*, BF# 210\NATIONAL COLL. OF INSECTS Pretoria, South Africa\ *Cis westerncapensis* Souza-Gonçalves & Lopes-Andrade HOLOTYPUS [red paper]”. Paratypes: 39 ♂♂ and 56 ♀♀ as follows: 1 ♀ (SANC) same data as the holotype; 2 ♀♀ (SANC) “SOUTH AFRICA WCAPE, Montagu Pass N of George, 33°54’ S 22°24’ E, 4.xi.2009, S & OC Neser\Ex bracket fungus, #211 on *Brachylaena neriifolia*\Ex bracket fungus *Coriolus hirsutus*, BF# 211”; 2 ♀♀ (CELC) “SOUTH AFRICA WCAPE, Montagu Pass N of George, 33°54’ S 22°24’ E, 4.xi.2009, S & OC Neser\Ex bracket fungus, #201 on fallen tree trunk\Ex bracket fungus *Ganoderma applanatum*, BF# 201\NATIONAL COLL. OF INSECTS Pretoria, South Africa”; 1 ♂ (CELC, dissected) and 1 ♀ (CELC) “SOUTH AFRICA WCAPE, Montagu Pass N of George, 33°54’ S 22°24’ E, 4.xi.2009, S & OC Neser\Ex bracket fungus, #199 on *Rapanea* sp.\Ex bracket fungus *Ganoderma applanatum*, BF# 199\NATIONAL COLL. OF INSECTS Pretoria, South Africa”; 1 ♂ and 1 ♀ (SANC) “SOUTH AFRICA, CP, Grootvadersbosch, nr. Heidelberg, 33.50S 20.53E, v.1985, A.J. Prins \ NATIONAL COLL. OF INSECTS Pretoria, S. Afr.”; 1 ♂ and 3 ♀♀ (CELC) “SOUTH AFRICA WCAPE, Prince Alfred’ S N of Knysna, 33°58’ S 23°09’ E, 5.xi.2009, S & OC Neser\Ex bracket fungus, #215 on fallen tree trunk\Ex bracket fungus *Fomitopsis lilacino-gilva*, BF# 215\NATIONAL COLL. OF INSECTS Pretoria, South Africa”; 1 ♀ (SANC) “SOUTH AFRICA WCAPE, Montagu Pass N of George, 33°54’ S 22°24’ E, 4.xi.2009, S & OC Neser\Ex bracket fungus *Coriolus hirsutus*, BF# 186\NATIONAL COLL. OF INSECTS Pretoria, South Africa”; 1 ♀ (SANC) “SOUTH AFRICA WCAPE, Montagu Pass N of George, 33°54’ S 22°24’ E, 4.xi.2009, S & OC Neser\Ex bracket fungus, #188 on fallen tree trunk\Ex bracket fungus *Coriolus* sp., BF# 188\NATIONAL COLL. OF INSECTS Pretoria, South Africa”; 3 ♂♂ (1 CELC, dissected; 2 SANC) and 3 ♀♀ (1 CELC, dissected; 2 SANC) “SOUTH AFRICA: WCape, Saasveld Foretry. Coll., 33°57’ S 22°32’ E, 14.viii.1990, AAJ Hendricks\Collected from rotten log of *Olea capensis* OLEACEAE, UA627\NATIONAL COLL. OF INSECTS Pretoria, South Africa”; 1 ♂ and 1 ♀ (SANC) “SOUTH AFRICA: WCape, Groeneweide Forest Walk, Saasveld, 33°58’ S 22°32’ E, 20.iii.1991, AJ Hendricks\Emerged from dead branches of living tree of *Rapanea melanophloeos* MYRSINIACEAE, UA716\NATIONAL COLL. OF INSECTS Pretoria, South Africa”; 2 ♂♂ (1 CELC; 1 SANC) “SOUTH AFRICA WCAPE, Prince Alfred’ S Pass N of Knysna, 35°58’ S 23°09’ E, 5.xi.2009, S & OC Neser\Ex bracket fungus, #219 on fallen tree trunk\NATIONAL COLL. OF INSECTS Pretoria, South Africa”; 24 ♂♂ (8 CELC; 16 SANC, 13 in dried capsule) and 31 ♀♀ (10 CELC; 21 SANC, 13 in dried capsule) “SOUTH AFRICA WCAPE, Montagu Pass N of George, 33°54’ S 22°24’ E, 4.xi.2009, S & OC Neser\Ex bracket fungus, #208 on fallen tree trunk\NATIONAL COLL. OF INSECTS Pretoria, South Africa”; 2 ♀♀ (SANC) “SOUTH AFRICA: WCAPE, Heatherlands, George, 33°57’ S 22°27’ E, 11.iv.1990, AJ Urban\Emerged from *Dais cotinifolia* THYMELAEACEAE, with fruiting body of fungus ?*Lenzites*, UA601”; 3 ♂♂ (1 CELC; 2 ANIC) and 5 ♀♀ (1 CELC; 4 ANIC) “REP. S. AFRICA: Cape Prov., Knysna, Diepwalle 450m, 17 Dec. 1981, 81-170, forest fungi & litter, S. & J. Peck, coll.”; 2 ♂♂ and 1 ♀ (ANIC) “REP. S. AFRICA: Cape Prov., Knysna, Gouna, 81-186, 23 Dec. 1981, berlese forest log litter, S. & J. Peck, coll.”; 1 ♂ and 1 ♀ (CELC) “REP. S. AFRICA: Cape Prov., Tsitsikama For. N.P., 20 Dec. 1981, 81-176, Berlese forest litter, S. & J. Peck, coll.”. All paratypes additionally labeled “*Cis westerncapensis* Souza-Gonçalves & Lopes-Andrade PARATYPUS [yellow paper]”.

**Host fungi:***Ganoderma applanatum* (Pers.) Pat., two records; *Rhodofomitopsis lilacinogilva* (Berk.) (Berk.) B.K. Cui, M.L. Han & Y.C. Dai (Fomitopsidaceae), one record; *Trametes* sp., one record; *Trametes hirsuta* (Wulfen) Lloyd, three records.

**Distribution:** Ethiopian. Known from southern Western Cape (Figure 13).

**Comments:** This species was collected together with *C. neserorum*, *C. parvisetosus* **sp. n.**, *C. regius*, the morphospecies *Orthocis* sp. A, *X. madagascariensis*, the invasive species *Cer. tabellifer*, and the parasitoids *A. gracilis* and *A. naiadis*.

### 3.2. Key to southern African species of Cis Latreille

Note: This identification key applies only to species described [1,9,10,11,12] or examined by us. The following southern Africa *Cis* species were not included: *C. afer*, *C. bimucronatus*, *C. caffer*, *C. capensis*, *C. delagoensis*, *C. muriceus* and *C. testaceus*. The key works for both males and females, except for *C. fuscipes*, of which only females are known to occur in southern Africa, and *C. aster* and *C. umlalaziensis* sp. n., of which only males are known.

1 Anterocephalic edge rounded, truncate, or projected in a single elevated plate or paired plates, horns, teeth or small angulations AND first abdominal ventrite with sex patch; IF sex patch absent, THEN both anterocephalic edge and anterior pronotal edge with short projections or subtriangular plates. Protibia with outer apical angle ALWAYS projected in an acute tooth. Males...........................................………………………………………………………………………………**2**1’ Anterocephalic edge truncate or barely emarginate AND first abdominal ventrite without sex patch. Protibia with outer apical angle variable, simple, projected in an acute tooth or bearing sockted spines. Females……………………….. ……………………………………………………………………………….**23**2 (1) Anterocephalic edge ALWAYS elevated and produced forming four teeth AND anterior pronotal edge projected in two small plates; IF pronotum with anterior edge simple, THEN head with occipital tubercle close to vertex….………………………………………….………………..………**3**2’ Anterocephalic edge NEVER elevated and produced forming four teeth (can be elevated and produced forming two teeth, but NEVER four teeth). Pronotum with anterior edge variable……………………….. ……………………………………………………………………………….**6**3 (2) Head with occipital tubercle close to vertex. Pronotum with anterior edge rounded; punctation dual. Elytral vestiture single, consisting of seriate setae. Tegmen with apex subtriangular delimited by internal excavations; penis with apex slightly enlarged and membranous. Southern WC……………………………………………….…….…….…….***Cis regius* Orsetti et Lopes-Andrade**3’ Head without occipital tubercle. Pronotum with anterior edge projected in two small plates; punctation single or inconspicuously dual. Elytral vestiture single, consisting of non-seriate bristles; IF vestiture dual, THEN consisting of mixed bristles and setae…………………………………………**4**4 (3’) Prosternum biconcave. Elytral vestiture dual, consisting of mixed short bristles and minute setae. Tegmen with apex bearing small emargination and globular tubercles in apical portion (Figure 10(H); black arrows); penis with apical portion membranous and bearing acute sclerotization at middle (Figure 10(I); big red arrow), and base with truncate sclerotization (Figure 10(I); small red arrows). Eastern and southeastern MP…………………***Cis tessariplacus* Souza-Gonçalves et Lopes-Andrade sp. n.**4’ Prosternum almost flat to tumid at midline. Elytral vestiture single, consisting of non-suseriate bristles……...…………...…..…..…..…..…..…..…..…..…..…..…..…..…..…..…..…..…..…..…..…..…..…..**5**5 (4’) Pronotum with punctures separated by less than one puncture-width; lateral margins barely to completely visible from above. Elytra with single punctation. Tegmen deeply and broadly emarginate at apex forming three lobes, the lateral ones barely acute and the mid one somewhat arrow-shaped; penis with basal third bearing lateral struts, which join to form membranous apex. Cosmopolitan; introduced at southern WC..………………………………..…..***Cis chinensis* Lawrence**5’ Pronotum with punctures separated by one to two puncture-widths; lateral margins not visible from above. Elytra with inconspicuously dual punctation. Tegmen deeply and broadly emarginate at apex forming three lobes, the lateral ones barely acute and the mid one somewhat arrow-shaped; penis with base bearing two short sclerotizations. Eastern and southeastern KZN….…………………………..…..…..…..……...***Cis paraliacus* Souza-Gonçalves et Lopes-Andrade**6 (2’) Elytral vestiture indistinctly to distinctly dual, IF indistinctly dual, THEN anterior pronotal edge barely to strongly emarginate forming short prominences or subtriangular plates.......................**7**6’ Elytral vestiture single and anterior pronotal edge ALWAYS rounded….…….…..…..…….……...**16**7 (6) Anterocephalic edge and anterior pronotal edge with one plate........…………..…..…..….....…….**8**7’ Anterocephalic edge and anterior pronotal edge barely to strongly emarginate, forming two short prominences or subtriangular plates……………………………………..…..…..…..…..…..…..…..….…..**9**8 (7) TL more than 1.30 mm. Anterocephalic edge with rounded corners. Pronotum with plate slightly emarginate forming two short projections with subrounded apex. Male with abdominal sex patch about one-third the length of first ventrite at midline. Tegmen bearing deep emargination at apex; penis subcylindrical and with apex bearing shallow emargination. Southern WC…………….................................................***Cis pickeri* Lopes-Andrade, Matushkina, Buder et Klass**8’ TL less than 1.30 mm. Anterocephalic edge with acute corners. Pronotum with plate angularly emarginate forming two small and triangular horns with acute apex. Male with abdominal sex patch about one-fourth the length of first ventrite at midline. Tegmen with apex bearing a small emargination on both sides; penis subcylindrical and with apex bearing short emargination. Northern and southern MP..…………..………...***Cis mooihoekite* Souza-Gonçalves et Lopes-Andrade**9 (7’) First male abdominal ventrite devoid of sex patch……………………………………..……...…...**10**9’ First male abdominal ventrite bearing sex patch ………………...……………………..…..…..……...**12**10 (9) Prosternal process relatively narrow, at least 4× as long as wide, no more than 0.15 as wide as gula. Tegmen elongate, with bilobed apex and membranous apical portion (Figure 8; black arrows); penis with three acute angulations at apex (Figure 9; red arrows). Northern MP, southeastern LP and northeastern NW…………………..…….***Cis bicaesariatus* Souza-Gonçalves et Lopes-Andrade sp. n.**10’ Prosternal process relatively wide, no more than 3× as long as wide, more than 0.20 as wide as gula……………………………………………........................…..…..…..…....…..…..…..…..…..…..….......**11**11 (10’) Pronotum with anterior portion bearing concave impression; IF concave impression is inconspicuous, THEN head visible from above. Prosternal process with rounded apex. Tegmen elongate, devoid of lateral excavations or angulations, and apical portion with narrow emargination at middle; penis subcylindrical. EC, GP, KZN, LP, MP, NW and WC………………..…..***Cis neserorum* Souza-Gonçalves et Lopes-Andrade**11’ Pronotum with anterior portion devoid of concave impression AND head not visible from above. Prosternal process with rounded apex. Tegmen elongate, somewhat mace-shaped, with each lateral edge hardened near middle and bearing small concave emargination with acute angulation at base; penis very elongate………………………..……………...***Cis aster* Souza-Gonçalves et Lopes-Andrade**12 (9’) Prosternal process conspicuously narrow near base and gradually expanding to rounded apex………………………………………….………………………………..…..…..…..…..…..…..…..…...**13**12’ Prosternal process subparallel-sided with rounded apex....………………..…..…..…..……………..**14**13 (12) Body very robust. Lateral pronotal margins smooth. Male with first abdominal ventrite bearing glabrous sex patch. Tegmen elongate, devoid of lateral excavations or angulations, apical portion with membranous areas and rounded sclerotization; penis subcylindrical. Eastern MP………………..…..…..…..…..…..…....…..…….....***Cis urbanae* Souza-Gonçalves et Lopes-Andrade**13’ Body not conspicuously robust. Lateral pronotal margins weakly crenulate. Male with first abdominal ventrite bearing setose sex patch. Tegmen with three deep emarginations at apex; penis with apical portion membranous and with V-shaped sclerotization. Eastern NW, western GP, eastern MP and northern LP…………………….…***Cis mandelai* Souza-Gonçalves et Lopes-Andrade**14 (12’) Anterocephalic edge with two triangular plates separated from each other by less than half width of scutellar shield and with rounded apices. Pronotum with anterior portion bearing concave impression. Male with first abdominal ventrite with minute sex patch, about one-tenth the length of first ventrite at midline. Tegmen with apical portion emarginated forming two lateral lobes with rounded apices, inner edges close to each other; penis with struts not linked at base. Eastern NW, eastern and western KZN………………..…...…………***Cis stalsi* Souza-Gonçalves et Lopes-Andrade**14’ Anterocephalic edge with two triangular plates separated from each other by one width of scutellar shield or a bit less, and with subacute to acute apices. Pronotum with anterior portion devoid of concave impression. Male with first abdominal ventrite with sex patch at middle, more than one-fifth the length of first ventrite at midline……...…..…..…..…..…..…..…..…..…..…..…..…...**15**15 (14’) Pronotum with anterior pronotal edge projected into two short triangular plates; apex, subacute; interspaces, barely microreticulate; anterior corners, rounded. Tegmen with apical portion bearing sclerotization at middle and laterals, which give the appearance of a trident; penis with two struts curved inwardly. Northeastern KZN and southeastern LP………………………….***Cis makebae* Souza-Gonçalves et Lopes-Andrade**15’ Pronotum with anterior edge emarginated anteriorly, forming two short projections; interspaces, coarsely microreticulate; anterior corners, angulate. Tegmen with membranous apical portion and bearing emargination at apex; penis with struts not linked at base and forming conspicuous sclerotizations, apex subtriangular and membranous. Eastern NW, northern LP and eastern MP………..………………………..……...…..…..….***Cis masekelai* Souza-Gonçalves et Lopes-Andrade**16 (6’) Elytra with punctation more or less to distinctly seriate………………………..…..…..………..**17**16’ Elytra with punctation non-seriate…………………………………..……...………..…..…..………...**20**17 (16) Lateral pronotal margins completely visible from above. Elytra with seriate and dual punctation; megapunctures forming more or less longitudinal rows, in-between rows filled with micropunctures; vestiture consisting of moderately short bristles arising from micropuntures.....…**18**17’ Lateral pronotal margins not visible from above. Elytra with subseriate and dual punctation; megapunctures not forming longitudinal rows; vestiture consisting of moderately short bristles arising from megapunctures....……………………………………...…..…..…..…..…..…..…..…..…….....**19**18 (17) Body very elongate and distinctly flattened. Anterocephalic edge truncate. Prosternum moderately long and flattened. Elytral vestiture consisting of short bristles (~0.01 mm). Tegmen with apex bearing one shallow emargination in each side and with acute angulations (Figure 6(G); big black arrows) and rounded corners (Figure 6(G); small black arrows); penis with rounded apex and with one excavation in each side (Figure 6(H); red arrows). Eastern MP and southeastern LP……***Cis makrosoma* Souza-Gonçalves et Lopes-Andrade sp. n.**18’ Body elongate and convex. Anterocephalic edge with small angulations. Prosternum concave. Elytral vestiture consisting of moderately short bristles (0.03–0.04 mm). Tegmen with apex bearing one deep emargination in each side and with acute angulations at corners (Figure 4(H); black arrows); penis with apical portion membranous and with a sclerotization at middle, apex truncate (Figure 4(I); red arrows). Northern MP and northwestern LP….…………….***Cis grobbelaarae* Souza-Gonçalves et Lopes-Andrade sp. n.**19 (17’) Color dark reddish brown. Elytral vestiture distinctly seriate, dense and short (0.02–0.03 mm). Male with first abdominal ventrite bearing unmargined sex patch. Tegmen with apex bearing rounded emargination and two small tubercles at middle (Figure 5(G); black arrows); penis with apex rounded and base with one flap in each side (Figure 5(H); red arrows). Western KZN and northern FS………………………..….….….….…….***Cis lacinipennis* Souza-Gonçalves et Lopes-Andrade sp. n.**19’ Color reddish brown. Elytral vestiture subseriate, sparse and long (0.03–0.04 mm). Male with first abdominal ventrite bearing margined sex patch. Tegmen with apex bearing two acute angulations in each side (Figure 12(H); black arrows); penis about 4× as long as tegmen and somewhat stick-shaped. Southern WC………………………....***Cis westerncapensis* Souza-Gonçalves et Lopes-Andrade sp. n.**20 (16’) Head with vertexal sex patch; anterocephalic edge with two small triangular tubercles. Tegmen with one rounded emargination in each side forming three lobes, the lateral ones short and acute (Figure 2(H); big black arrows), the mid one long and somewhat arrow-shaped (Figure 2(H); small black arrow); penis elongate and with membranous apex. Southeastern MP, northeastern NW and western KZN……………………………***Cis foveocephalus* Souza-Gonçalves et Lopes-Andrade sp. n.**20’ Head ALWAYS without vertexal sex patch; anterocephalic edge with two subtriangular or triangular plates……………………………………………...………….….….….….….….….….….….….**21**21 (20’) Anterocephalic edges with plates longer than scutellar shield at midline. Elytral vestiture subseriate. Tegmen with apex bearing one emargination in each side, forming three rounded lobes (Figure 11(F); black arrows), the lateral ones slightly shorter than the mid one; penis subcylindrical and with subtriangular apex. Eastern KZN…………………………***Cis umlalaziensis* Souza-Gonçalves et Lopes-Andrade sp. n.**21’ Anterocephalic edges with plates shorter than scutellar shield at midline. Elytral vestiture non-subseriate..……………………………………………….….……..….….….….….….….….…...….…**22**22 (21’) Dorsal vestiture consisting of thin and sparse setae. Tegmen apex bearing shallow somewhat V-shaped emargination and with rounded corners (Figure 9(B); black arrows); penis subcylindrical and with subtriangular apex. Southern WC, southeastern and western KZN…….....................................……….....***Cis parvisetosus* Souza-Gonçalves et Lopes-Andrade sp. n.**22’ Dorsal vestiture consisting of thick and dense bristles. Tegmen with apex bearing deep V-shaped emargination, forming lateral struts curved to middle (Figure 7(H); black arrows); penis subcylindrical and with triangular apex. Eastern and southeastern MP, northern and southeastern LP…………………….….….…….…***Cis mpumalangaensis* Souza-Gonçalves et Lopes-Andrade sp. n.**23 (1’) Elytra with punctation more or less to distinctly seriate…….............…….….….……………….**24**23’ Elytra with punctation non-seriate………………………………….…………..….….…..…………...**28**24 (23) Lateral pronotal margins completely visible from above. Elytral vestiture consisting of short or moderately long bristles arising from micropunctures….……….……...….….….….….….….…..…**25**24’ Lateral pronotal margins not visible from above. Elytral vestiture consisting of short bristles arising from megapunctures……………………………….….….….….….…...….….….….….…………**27**25 (24) Protibia with outer apical angle dentate. Cosmopolitan; introduced at southeastern MP and southern WC………………………………....….….….….….….….….….….……..…...***Cis fuscipes* Mellié**25’ Protibia with outer apical angle simple..……………………………….….…....….….….…………...**26**26 (25’) Body very elongate and distinctly flattened. Prosternum moderately long and flattened. Elytral vestiture consisting of short bristles (~0.01 mm). Eastern MP and southeastern LP…...………………………………….…...***Cis makrosoma* Souza-Gonçalves et Lopes-Andrade sp. n.**26’ Body elongate and convex. Prosternum concave. Elytral vestiture consisting of moderately short bristles (0.03–0.04 mm). Northern MP and northwestern LP…………………………...***Cis grobbelaarae* Souza-Gonçalves et Lopes-Andrade sp. n.**27 (24’) Color dark reddish brown. Elytral vestiture dense, short (0.02–0.03 mm), distinctly seriate. Western KZN and northern FS….…….....***Cis lacinipennis* Souza-Gonçalves et Lopes-Andrade sp. n.**27’ Color reddish brown. Elytral vestiture subseriate, sparse and long (0.03–0.04 mm). Southern WC……………..…………..………….***Cis westerncapensis* Souza-Gonçalves et Lopes-Andrade sp. n.**28 (23’) Elytral vestiture distinctly dual; IF indistinctly dual, THEN punctation dual..………………**29**28’ Elytral vestiture single……………………………………………………………...................................**39**29 (28) Protibia with outer apical angle expanded and bearing socketed spines....................................**30**29’ Protibia with outer apical angle not expanded and devoid of spines……………………...………..**31**30 (29) TL more than 1.30 mm. Southern WC….……………..…………….................................***Cis pickeri* Lopes-Andrade Matushkina, Buder et Klass**30’ TL less than 1.30 mm. Northern and southern MP..………..…………………………***Cis mooihoekite* Souza-Gonçalves et Lopes-Andrade**31 (29’) Elytra with sigle punctation. Eastern and southeastern MP………….……...***Cis tessariplacus* Souza-Gonçalves et Lopes-Andrade sp. n.**31’ Elytra with dual punctation …………………………………………………………………………….**32**32 (31’) Prosternal process conspicuously narrow near base and gradually expanding to rounded apex…..…………………………………………………………………………………………….………….**33**32’ Prosternal process subparallel- or parallel-sided, with rounded or truncate apex….….………….**35**33 (32); Body very robust. Lateral pronotal margins smooth Eastern MP…………………..***Cis urbanae* Souza-Gonçalves et Lopes-Andrade**33’ Body not conspicuosly robust. Lateral pronotal margins weakly crenulate. Eastern NW, western GP, eastern MP and northern LP…………………..***Cis mandelai* Souza-Gonçalves et Lopes-Andrade**35 (32’) Prosternal process relatively narrow, at least 4× as long as wide, no more than 0.15 as wide as gula. Northern MP, southeastern LP and northeastern NW…………..……………..***Cis bicaesariatus* Souza-Gonçalves & Lopes-Andrade sp. n.**35’ Prosternal process relatively wide, no more than 3× as long as wide, more than 0.20 as wide as gula………………………………………………………………………………………….............................**36**36 (35’) Pronotal punctures usually separated by more than one puncture diameter and interspaces barely microreticulate. Northeastern KZN and southeastern LP.......……..………………..***Cis makebae* Souza-Gonçalves et Lopes-Andrade**36’ Pronotal punctures usually separated by less than one puncture-width and interspaces coarsely microreticulate………………………………………………………………………………………..………**37**37 (36’) Pronotum with anterior corners angulate. Eastern NW, northern LP and eastern MP……………………..………………………………………***Cis masekelai* Souza-Gonçalves et Lopes-Andrade**37’ Pronotum with anterior corners rounded……………………..………………………………………**38**38 (37’) Scutellar shield about 2× as long as elytral megapunctures. Eastern NW, eastern and western KZN…………………....................................………….…***Cis stalsi* Souza-Gonçalves et Lopes-Andrade**38’ Scutellar shield about 4× as long as elytral megapunctures. EC, GP, KZN, LP, MP, NW and WC…………………………...….…...………………***Cis neserorum* Souza-Gonçalves et Lopes-Andrade**39 (28’) TL usually less than 1.80 mm; IF about 1.80 mm, THEN elytral vestiture consisting of short bristles (0.01–0.02 mm)...………………………………………………...…………………………………..**40**39’ TL more than 1.80 mm AND elytral vestiture consisting of moderately short to moderately long bristles (at least 0.04 mm)………..……………………….…………………...……………………………..**43**40 (39) Elytral vestiture seriate, consisting of moderately long setae (0.04–0.05 mm). Southern WC…………………………………………………………………….***Cis regius* Orsetti et Lopes-Andrade**40’ Elytral vestiture non-seriate, consisting of short bristles or minute setae (≥0.02 mm)………...…..**41**41 (40’) Pronotum devoid of impunctate median line; punctures separated by two puncture-widths or less; lateral margins crenulate. Southeastern MP, northeastern NW and western KZN………………………………...……***Cis foveocephalus* Souza-Gonçalves et Lopes-Andrade sp. n.**41’ Pronotum bearing impunctate median line; punctures usually separated by one puncture -width or less; lateral margins not crenulate………………………………...……………………………………..**42**42 (41’) Dorsal vestiture consisting of thin and sparse setae. Southern WC, southeastern and western KZN……...................................…..……….***Cis parvisetosus* Souza-Gonçalves et Lopes-Andrade sp. n.**42’ Dorsal vestiture consisting of thick and dense bristles. Eastern and southeastern MP, northern and southeastern LP……..………...***Cis mpumalangaensis* Souza-Gonçalves et Lopes-Andrade sp. n.**43 (39’) Pronotum with punctures separated by less than half a puncture-width; lateral margins barely to completely visible from above. Elytra with single punctation. Cosmopolitan; introduced at southern WC..…………..…..………….……..…………..…..…………..…..…..…***Cis chinensis* Lawrence**43’ Pronotum with punctures separated by one to two puncture-widths; lateral margins not visible from above. Elytra with inconspicuously dual punctation. Eastern and southeastern KZN….….………………………..…………………***Cis paraliacus* Souza-Gonçalves et Lopes-Andrade**

## 4. Discussion

The continental and insular sub-Saharan Ciidae faunas are more diverse than the northern fauna. The northern African fauna encompass 14 described species in four genera: *Cis* (10 species), *Hadreule* Thomson (1), *Orthocis* Casey (1) and *Ropalodontus* Mellié (2); and most of these species, if not all, are shared with the European fauna. The continental and insular sub-Saharan faunas encompass 86 described species in eight genera: *Ceracis* Mellié (1 introduced species), *Cis* (63, of which 11 were added in the present work), *Dimerapterocis* Scott (1), *Ennearthron* Mellié (1), *Orthocis* (6), *Paratrichapus* Scott (1), *Tropicis* Scott (4) and *Xylographus* Mellié (9).

*Cis* is the most diverse genus in southern Africa (comprising Botswana, Lesotho, Namibia, Republic of South Africa, Swaziland and the southern tip of Mozambique), and the great majority of species are known to occur in one or few provinces of Republic of South Africa. *Cis neserorum* is the unique exception occurring in seven of nine South African provinces [11]. Besides *C. neserorum* that is distributed from the south throughout the northeast of Republic of South Africa, the other species are more distributed in the northeast, with just eigth species occurring only in the southeast (*C. afer*, *C*, *caffer*, *C. capensis*, *C. chinensis*, *C. muriceus*, *C. pickeri*, *C. regius* and *C. westerncapensis*) and two occurring in the south and northeast (*C. fuscipes* and *C. parvisetosus*) (Figure 13, Figure 14 and Figure 15) (see distributional maps in Souza-Gonçalves & Lopes-Andrade [11,12,13]). It is important to note that most southern African *Cis* are restricted to these areas.

The southern African ciid with the largest distribution is the invasive species *Cer. tabellifer* [48]. The distributional patterns of southern African *Cis* species could provide insights about the possible impacts of the invasive species *Cer. tabellifer* in the autochthonous fauna, a hypothesis under evaluation by us and to be treated in a forthcoming paper. However, we can already highlight that there is likely a displacement of autochthonous *Cis* species to the northeastern of the Republic of South Africa (Figure 13, Figure 14 and Figure 15) (see distributional maps in Souza-Gonçalves & Lopes-Andrade [11,12,13]).

Except for the South African fauna, of which knowledge has been increased in the last years [1,10,11,12,48], information about the southern African fauna as a whole is very poor. The available information for the rest of this area is restricted to the original descriptions only and designations of lectotypes [21,22,23,24,25,49], without any further records in the scientific literature. Recent field collections and examination of historical collections have resulted in new discoveries about the African fauna, as undescribed genera (one from Tanzania and another from Madagascar), and records of undescribed species of *Notapterocis* Lawrence and *Aliocis* Sandoval-Gómez & Lopes-Andrade from the Republic of South Africa, which are being described by us.

Some species have been redescribed [27] and new species have been found in historical collections [13]; however, a gap in the literature remains concerning species from Central Africa and some from southern Africa. In most described species, there is no information on the morphology of sclerites of male and female abdominal terminalia, and specially the morphology of male genitalia, which is crucial for defining species boundaries and identifying them.

## 5. Conclusions

The southern African *Cis* fauna, comprising Botswana, Lesotho, Namibia, Republic of South Africa, Swaziland and the southern tip of Mozambique, now encompass 29 species: *C. afer*, *C. aster*, *C. bicaesariatus*, *C. bimucronatus*, *C. caffer*, *C. capensis*, *C. chinensis*, *C. delagoensis*, *C. foveocephalus*, *C. fuscipes*, *C. grobbelaarae*, *C. lacinipennis*, *C. makebae*, *C. makrosoma*, *C. mandelai*, *C. masekelai*, *C. mooihoekite*, *C. mpumalangaensis*, *C. muriceus*, *C. neserorum*, *C. paraliacus*, *C. parvisetosus*, *C. regius*, *C. stalsi*, *C. tessariplacus*, *C. testaceus*, *C. urbanae*, *C. umlalaziensis* and *C. westerncapensis*. We conclude that examination of sclerites of male terminalia is the most accurate way for defining species boundaries and identifying them, mainly those of the *neserorum* and *pacificus* groups, of which the species are externally very similar to each other. The knowledge concerning the few Central African and southern Africa species described in the XIX and beginning of the XX centuries is very poor, and it is still necessary to examine type material and provide information and images, especially of their male genitalia.
